# Targeting Bacteria‐Induced Ferroptosis of Bone Marrow Mesenchymal Stem Cells to Promote the Repair of Infected Bone Defects

**DOI:** 10.1002/advs.202404453

**Published:** 2024-08-21

**Authors:** Kai Yuan, Yiqi Yang, Yixuan Lin, Feng Zhou, Kai Huang, Shengbing Yang, Weiqing Kong, Fupeng Li, Tianyou Kan, Yao Wang, Caiqi Cheng, Yakun Liang, Haishuang Chang, Jie Huang, Haiyong Ao, Zhifeng Yu, Hanjun Li, Yihao Liu, Tingting Tang

**Affiliations:** ^1^ Shanghai Key Laboratory of Orthopaedic Implants Department of Orthopaedic Surgery Shanghai Ninth People's Hospital Shanghai Jiao Tong University School of Medicine Shanghai 200011 P. R. China; ^2^ Department of Orthopedics The First Affiliated Hospital Zhejiang University School of Medicine 79 Qingchun Rd Hangzhou 310003 P. R. China; ^3^ Department of Orthopaedic Surgery The First Affiliated Hospital of Soochow University No. 899 Ping Hai Road Suzhou Jiangsu 215006 P. R. China; ^4^ Department of Orthopaedic Surgery Xuzhou Central Hospital Xuzhou Clinical School of Xuzhou Medical University 199 Jiefang South Road Xuzhou 221009 P. R. China; ^5^ Shanghai Institute of Precision Medicine Shanghai Ninth People's Hospital Shanghai Jiao Tong University School of Medicine Shanghai 200125 P. R. China; ^6^ Jiangxi Key Laboratory of Nanobiomaterials & School of Materials Science and Engineering East China Jiaotong University Nanchang 330000 P. R. China; ^7^ State Key Laboratory of Systems Medicine for Cancer Renji‐Med X Clinical Stem Cell Research Center Ren Ji Hospital Shanghai Jiao Tong University School of Medicine 160 Pujian Road Shanghai 200127 P. R. China

**Keywords:** 3D‐printed scaffold, BMSC, bone infections, ferroptosis, infected bone defects

## Abstract

The specific mechanisms underlying bacteria‐triggered cell death and osteogenic dysfunction in host bone marrow mesenchymal stem cells (BMSCs) remain unclear, posing a significant challenge to the repair of infected bone defects. This study identifies ferroptosis as the predominant cause of BMSCs death in the infected bone microenvironment. Mechanistically, the bacteria‐induced activation of the innate immune response in BMSCs leads to upregulation and phosphorylation of interferon regulatory factor 7 (IRF7), thus facilitating IRF7‐dependent ferroptosis of BMSCs through the transcriptional upregulation of acyl‐coenzyme A synthetase long‐chain family member 4 (ACSL4). Moreover, it is found that intervening in ferroptosis can partially rescue cell injuries and osteogenic dysfunction. Based on these findings, a hydrogel composite 3D‐printed scaffold is designed with reactive oxygen species (ROS)‐responsive release of antibacterial quaternized chitosan and sustained delivery of the ferroptosis inhibitor Ferrostatin‐1 (Fer‐1), capable of eradicating pathogens and promoting bone regeneration in a rat model of infected bone defects. Together, this study suggests that ferroptosis of BMSCs is a promising therapeutic target for infected bone defect repair.

## Graphical abstract

Ferroptosis significantly impacts the viability and osteogenic potential of bone marrow mesenchymal cells (BMSCs) in infected bone environments. *S. aureus* and *E. coli* activate Toll‐like receptor signaling and initiate IRF7‐dependent ferroptosis in BMSCs. This study highlights the potential of targeting ferroptosis to enhance bone regeneration in infected bone defects using a ROS‐responsive antibacterial/anti‐ferroptosis 3D‐printed scaffolds.

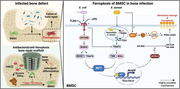

## 1 Introduction

Infected bone defects represent one of the most persistent and devastating consequences in orthopedic clinics. Patients with persistent infected bone disease suffer a high rate of pathological fractures, nonunion, limb deformities, and even death.^[^ ^1^ ^]^ Gram‐positive *Staphylococcus aureus* (*S. aureus*) is the most notorious and predominant pathogen in bone and joint infections (BJI), and Gram‐negative bacteria such as *Pseudomonas aeruginosa* (*P. aeruginosa*) and *Escherichia coli* (*E. coli*) also participate in BJI.^[^ ^2^ ^]^ The residual bacteria and chronic antimicrobial inflammation result in tissue injury and impede the normal osteogenic regenerative process, while stimulating osteoclast‐mediated bone resorption. Consequently, infected bone defects may ultimately develop into sequestrum formation, fibrous pseudo‐union, nonunion, or osteolytic bone lesions.^[^ ^3^ ^]^ Although antimicrobial treatments, such as debridement, antibiotics, and antibiotic‐PMMA beads, have greatly enhanced the effectiveness of treatment, the management of infected bone defects still confronts a high failure rate due to reasons such as incomplete eradication of bacteria and dysfunction of osteogenic regeneration.^[^ ^4^ ^]^ The underlying mechanisms of osteogenic dysfunction caused by bacteria remain unclear and developing an effective therapy for repairing infected bone defects is still an ongoing challenge that needs to be addressed.

Optimal bone defect regeneration and successful union rely on the abundant availability and highly orchestrated osteogenic differentiation of bone progenitor cells, including BMSCs and periosteum‐derived stem cells (PSCs).^[^ ^5^ ^]^ Previous studies showed that BMSCs take the main role in the repair of drilled bone injuries and PSCs become the primary cell type responsible for bone repair in bicortical fractures.^[^ ^6^ ^]^ Over the past decades, extensive research on bone infections revealed that toxins and metabolites secreted by pathological bacteria, along with various inflammatory factors and cytokines present in the infection microenvironment, collectively contribute to the injury of BMSCs and osteoblasts, leading to impairment of their osteogenic potential.^[^ ^7^ ^]^ For example, Staphylococcal protein A (SPA) and phenol‐soluble modulins (PSMs) could induce the death of osteoblast lineage cells.^[^ ^8^ ^]^ Immune activation by bacterial components or inflammatory factors also causes injury to BMSCs and hampers normal osteogenic differentiation.^[^ ^7^, ^9^ ^]^ However, these studies roughly attributed the death of infected BMSCs or osteoblasts to apoptosis or cell lysis, which lacks specific morphological and genetic evidence. In addition, recent studies emphasized the importance of intracellular survival of *S. aureus*, the predominant pathogen in BJI, in BMSCs and osteoblasts.^[^ ^10^ ^]^ Intracellular bacteria could adjust the expression and release of virulence factors to avoid cytolysis, which helps the bacteria escape the bactericidal effects of antibiotics.^[^ ^11^ ^]^ This type of intracellular infection makes the mechanisms of BMSC death more elusive and intriguing. The exact mechanisms of direct injury and cell death of BMSCs caused by live bacteria remain unclear.

Ferroptosis is a newly identified form of programmed cell death that is characterized by iron‐dependent lipid peroxidation.^[^ ^12^ ^]^ Recent evidence suggests that ferroptosis plays an important role in infection and bacteria‐related cell death. The ferroptosis of macrophages and bronchial epithelium cells could be induced by *Mycobacterium tuberculosis* (*M. tuberculosis*) and *P. aeruginosa*.^[^ ^13^ ^]^ Meanwhile, ferroptosis also served as a potent therapeutic target for infectious diseases. Pharmacological inhibition of ferroptosis by Fer‐1 treatment effectively alleviates pathological damage and bacteria burden in the lung of the *M. tuberculosis*‐infected model.^[^ ^13a^ ^]^ Therefore, targeting ferroptosis may also be an effective strategy to rescue BMSC function and promote infected bone defect repair.

Treatment of critical‐sized infected bone defects usually requires the application of bioactive bone repair materials in a two‐stage operation after complete clearance of residual pathogens.^[^ ^14^ ^]^ Recent advances shed light on the promising application of 3D‐printed scaffolds and antimicrobial hydrogel in infected bone defect repair.^[^ ^15^ ^]^ However, most materials release antimicrobial and bioactive factors at a constant velocity and lack the capability of infection‐responsive releasing, which could not adapt to the dynamic bacterial burden and lead to failure of early pathogen eradication and alleviation of tissue damage. There is still a lack of an effective strategy for protecting bone progenitor cells and reserving tissue osteogenic potential in early infection, owing to a lack of understanding of the mechanism of bacteria‐induced BMSC death. Long‐lasting release of bioactive factors in repair materials is needed to activate tissue regenerative potential and restore innate physiological homeostasis of regeneration in defect sites.

In this study, we established acute osteomyelitis models and implant‐associated bone infection models in vivo, as well as a BMSC‐bacteria co‐culture model in vitro. Ferroptosis was identified to play a vital role in the death and osteogenic dysfunction of BMSCs induced by *S. aureus* and *E. coli* infections. Additionally, we designed and developed a dual‐functional antimicrobial/anti‐ferroptosis 3D‐printed bone repair scaffold with ROS‐responsive bioactive factors‐releasing activities. This scaffold represents a novel strategy for repairing critical‐sized infected bone defects. These results provide insights into the underlying mechanisms of infected bone defects and offer a promising therapeutic approach for infected bone defects repair.

## 2 Results

### 2.1 *S. aureus* and *E. coli* Induced Lipid Peroxides Accumulation in Bone Marrow and BMSCs

**Figure 1:**
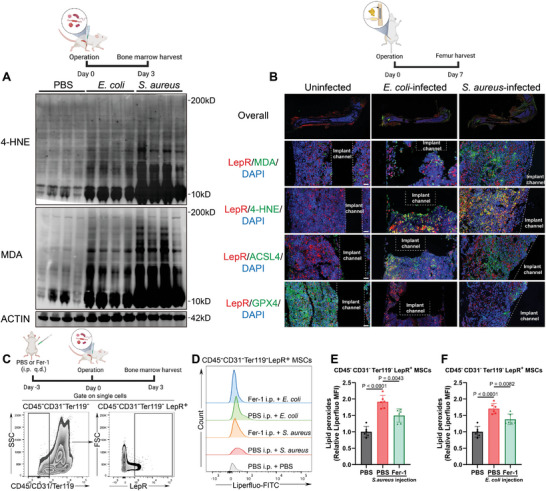
*S. aureus* and *E. coli* infections induced lipid peroxides accumulation in infected bone microenvironment and BMSCs. A) Lipid peroxidation levels in bone marrow on Day 3 post‐infection in murine osteomyelitis model induced by *S. aureus* and *E. coli* were determined by WB analysis of 4‐HNE and MDA protein modifications. Each group contained four mice. B) Representative tissue immunofluorescence images of LepR, MDA, 4‐HNE, ACSL4, GPX4 in the uninfected murine femur and implant‐associated bone infection model induced by *S. aureus* and *E. coli*. Scale bar = 50 µm. C) The schematic route of animal experiments and gating strategy were depicted. D) Lipid peroxide regulation by *S. aureus* and *E. coli* infections and Fer‐1 in CD45^−^CD31^−^Ter119^−^LepR^+^ BMSCs were analyzed in murine osteomyelitis model by Liperfluo staining and analyzed by flow cytometry. E,F) Quantification of median fluorescent intensity in flow cytometry analysis in (D). Values are means ± SDs. Each group contained six mice. Multiple comparison was performed by one‐way analysis of variance (ANOVA) with Tukey's post‐hoc analysis.

To elucidate the microenvironmental changes within infected bone tissues, we established murine models of acute osteomyelitis and implant‐associated bone infections using *S. aureus* and *E. coli*, primary causative pathogens in clinical bone infections. Lipid peroxidation levels in bone marrow cells were determined by measuring protein modifications of lipid peroxidation products, such as 4‐Hydroxynonenal (4‐HNE) and malondialdehyde (MDA) protein modifications. Initially, we assessed these protein modifications in bone marrow within the acute osteomyelitis model. As illustrated in **Figure** **1**A, in comparison to the uninfected mice, both 4‐HNE and MDA protein modifications were markedly elevated following *S. aureus* and *E. coli* exposure. This signifies a heightened lipid peroxidation within the infected bone marrow.

Given the tight association between lipid peroxidation and ferroptosis, we further examined protein‐lipid peroxidation modifications and markers of ferroptosis in the implant‐associated bone infection model. Tissue immunofluorescence staining (Figure 1B) revealed pronounced fluorescence signals for 4‐HNE and MDA protein modifications in the peri‐implant bone marrow cells 7 days post‐infection. These signals overlapped with leptin receptor positive (LepR^+^) cells, hinting at possible BMSCs involvement. Concurrently, we observed an increase of acyl‐CoA synthetase long‐chain family member 4 (ACSL4), an important contributor to ferroptosis, and a decrease of glutathione peroxidase 4 (GPX4), a key enzyme to prevent ferroptosis, within LepR^+^ cells.

To pinpoint lipid peroxide in BMSCs and ascertain the role of BMSCs ferroptosis in vivo within an infected bone environment, we used flow cytometry to analyze the bacterial induced lipid peroxide and the potential mitigative effects of the Fer‐1. Leptin receptor was recognized to be a reliable marker for BMSCs in adult mice, so CD45^−^CD31^−^Ter119^−^LepR^+^ cells in bone marrow were gated as BMSCs (Figure 1C) according to previous studies.^[^ ^16^ ^]^ Figure 1D–F depict a considerable increase of lipid peroxide in BMSCs subjected to *S. aureus* and *E. coli* intratibial injections (pre‐treated with phosphate buffered saline (PBS)) by 1.92 ± 0.20‐fold and 1.71 ± 0.16‐fold, respectively, compared to the PBS pre‐treated uninfected group (1.00 ± 0.16‐fold). Treatment with daily 10 mg kg^−1^ intraperitoneal Fer‐1 administration effectively alleviated the accumulation of lipid peroxides of BMSCs induced by *S. aureus* (1.50 ± 0.20‐fold) and *E. coli* (1.38 ± 0.16‐fold). These findings demonstrated that *S. aureus* and *E. coli* induced lipid peroxidation within the bone marrow and triggered ferroptosis in BMSCs in vivo.

### 2.2 *S. aureus* and *E. coli* Induced Ferroptosis of BMSCs In Vitro

While the accumulation of lipid peroxides in the infected bone microenvironment and ferroptosis in BMSCs has been identified, the primary mechanism driving cell injury and subsequent death in infected BMSCs remains ambiguous. To elucidate this question, we designed an in vitro BMSCs‐bacteria co‐culture model. We determined the optimal multiplicity of infection (MOI) to be 10 based on Live/Dead staining results (Figure S1, Supporting Information), ensuring an adequate number of cells for subsequent analysis.

**Figure 2:**
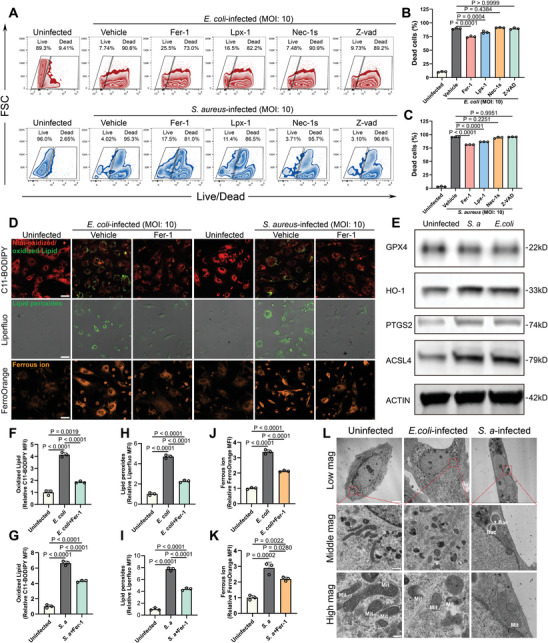
*S. aureus* and *E. coli* infections trigger ferroptosis in BMSCs in vitro. A) The rescuing effects of inhibitors array on *S. aureus* and *E. coli*‐induced BMSCs death was analyzed by Live/Dead staining assay and flow cytometry analysis. BMSCs were pretreated with various death inhibitors for 12 h and then infected with *S. aureus* (MOI: 10) and *E. coli* (MOI: 10) for 12 h before analysis. B,C) Quantification of the rate of dead cells in flow cytometry analysis in (A). Values are means ± SDs (*n* = 3). Multiple comparison was performed by one‐way analysis of variance (ANOVA) with Tukey's post‐hoc analysis. D) The levels of lipid peroxides and ferrous iron in uninfected or *S. aureus*‐ or *E. coli*‐infected BMSCs were determined by fluorescent staining of C11‐BODIPY, Liperfluo and FerroOrange. Representative fluorescent images of C11‐BODIPY staining (Scale bar = 40 µm), Liperfluo staining (Scale bar = 40 µm), and FerroOrange staining (Scale bar = 40 µm) were presented. E) WB analysis of the expression of GPX4, HO‐1, PTGS2, and ACSL4 in BMSCs. Quantification of fluorescent intensity of oxidized lipid F,G), lipid peroxides H,I), and ferrous iron J,K). Values are means ± SDs (*n* = 3). Multiple comparison was performed by one‐way analysis of variance (ANOVA) with Tukey's post‐hoc analysis. L) Representative TEM images of BMSCs in the uninfected group, *S. aureus*‐infected, and *E. coli*‐infected groups with different magnification degrees. Scale bar = 5 µm for low magnification, 750 nm for middle magnification, and 250 nm for high magnification. Mitochondria was abbreviated as Mit, and bacteria was abbreviated as Bac.

Utilizing flow cytometry (**Figure** **2**A–C), we found that among the array of death inhibitors, including inhibitors targeting ferroptosis (Fer‐1 and Liproxstatin‐1 (Lpx‐1)), necroptosis (Nec‐1S), apoptosis, and pyroptosis (Z‐VAD‐FMK), only ferroptosis inhibitors considerably reduced the mortality rate of BMSCs infected with *S. aureus* and *E. coli*. To rule out any antimicrobial activity of these inhibitors, we conducted antimicrobial assays. The results confirmed no significant antimicrobial properties of these inhibitors at specified concentrations (Figure S2, Supporting Information), emphasizing the pivotal role of ferroptosis in bacterial‐induced BMSC injury.

Ferroptosis is typically characterized by augmented levels of lipid peroxides and a surge in intracellular ferrous ion accumulation. To quantify these markers, we utilized specific fluorescent probes: C11‐BODIPY and Liperfluo for lipid peroxides, and FerroOrange for ferrous ions. Intriguingly, Both *E. coli* and *S. aureus* infections robustly enhanced these markers, with Fer‐1 treatment effectively mitigating these increments (Figure 2D,F–K). Quantitative analysis of fluorescence intensity (Figure 2F,G) showed that levels of oxidized lipid stained by C11‐BODIPY in BMSCs increased to 4.15 ± 0.18‐fold (*E. coli*‐infected) and 6.64 ± 0.28‐fold (*S. aureus*‐infected) compared with that of uninfected BMSCs (1.00 ± 0.21‐fold). And accumulation of ferrous ions in BMSCs increased to 3.39 ± 0.14‐fold in the *E. coli*‐infected group and 2.89 ± 0.37‐fold in the *S. aureus*‐infected group, which was mitigated by Fer‐1 administration (Figure 2J,K).

Further investigation into the expression patterns of ferroptosis‐related proteins revealed that while *S. aureus* and *E. coli* infections downregulated the ferroptosis‐suppressing protein GPX4, yet simultaneously elevated the levels of ferroptosis‐promoting proteins such as ACSL4, heme oxygenase (HO‐1), and Prostaglandin‐endoperoxide synthase 2 (PTGS2) (Figure 2E). Transmission electron microscopy (TEM) (Figure 2L) provided morphological evidence corroborating ferroptosis induction in infected BMSCs. Typical ferroptosis features such as the smaller size of the mitochondrion and the destroyed structure of mitochondrial cristae were observed. Additionally, intracellular bacteria were observed in *S. aureus*‐infected BMSCs, but not in *E. coli*‐infected BMSCs, hinting at different signaling pathway underlying the pathogen‐induced ferroptosis.

Combining the findings from the mortality rescue trials, lipid peroxide and ferrous ion fluorescence analysis, and molecular and morphological characterizations, we proposed that ferroptosis is the principal cell death mechanism in *S. aureus*‐ and *E. coli*‐infected BMSCs, potentially contributing to osteogenic dysfunction.

### 2.3 RNA‐Sequencing of BMSCs Infected by *S. aureus* and *E. coli*

**Figure 3:**
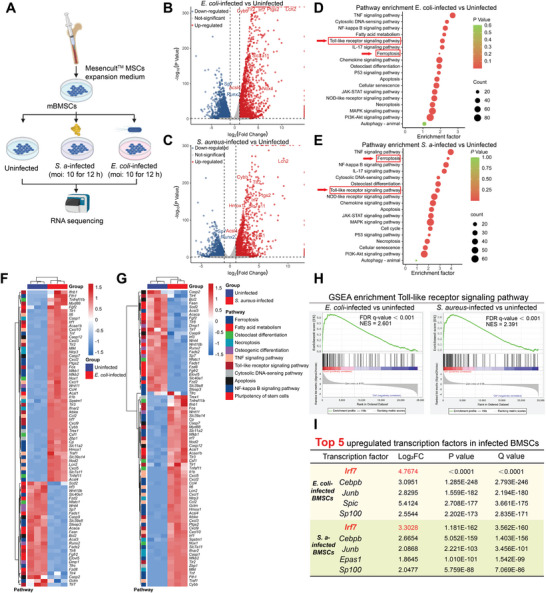
*S. aureus* and *E. coli* infections activated ferroptosis and toll‐like signaling pathways in BMSCs. A) Schematic illustration of BMSCs‐bacteria co‐culture model. RNA samples of uninfected BMSCs and BMSCs infected with *S. aureus* (MOI: 10 for 12 h) and *E. coli* (MOI: 10 for 12 h) were collected for RNA‐sequencing analysis. Volcano plot of differentially expressed genes (DEGs) in *E. coli* ‐infected BMSCs B) and *S. aureus*‐infected BMSCs C) compared with uninfected BMSCs. A fold change of protein (FC) ≥ 1.2‐fold and Q value < 0.05 were considered as DEPs. Upregulated DEGs are labeled with red dots, and downregulated DEGs are labeled with blue dots. Gray dots indicate nonsignificant differentially changed proteins. Some important DEGs related to ferroptosis and osteogenic differentiation were marked with gene names. KEGG pathway enrichment analysis of DEGs in *E. coli*‐infected BMSCs D) and *S. aureus*‐infected BMSCs E). Some DEGs related to different biological functions in *E. coli*‐infected BMSCs F) and *S. aureus*‐infected BMSCs G) were visualized by heatmap. H) GSEA enrichment of toll‐like receptor signaling pathway in *E. coli*‐infected BMSCs (Enrichment score = 0.659 and FDR Q value < 0.001) and *S. aureus*‐infected BMSCs (Enrichment score = 0.647 and FDR Q value < 0.001). I) Top 5 upregulated transcription factors in *E. coli*‐infected BMSCs and *S. aureus*‐infected BMSCs.

To unveil the underlying mechanisms of infection‐triggered ferroptosis in BMSCs, RNA‐sequencing analysis was performed. As depicted in **Figure** **3**A, uninfected BMSCs and BMSCs infected by *S. aureus* (MOI:10 for 12 h) and *E. coli* (MOI:10 for 12 h) were collected and subjected to RNA‐sequencing. The principal component analysis (PCA) (Figure S3A, Supporting Information) underscored the consistency in gene expression patterns within individual treatment groups. Moreover, it highlighted distinct differences in the gene expression patterns between the three treatment groups. Indeed, *E. coli* and *S. aureus* triggered intense transcriptional changes. As depicted in volcano plots (Figure 3B,C), compared with uninfected BMSCs, a total of 3624 differentially expressed genes (DEGs) were identified in *E. coli*‐infected BMSCs, with 1149 DEGs upregulated and 2475 DEGs downregulated. As for *S. aureus*‐infected BMSCs, 866 upregulated DEGs and 1029 downregulated were identified compared with uninfected BMSCs. Among DEGs (Figure 3F,G), we observed significant downregulation of pivotal genes such as *Runx2*, *Sp7*, *Wnt4*, *Fzd2*, *Dmp1*, and ferroptosis‐suppressing such as *Acsl3* in *E. coli*‐infected and *S. aureus*‐infected BMSCs, which correlated with osteogenic dysfunction in infected BMSCs. Meanwhile, DEGs indicate that pathways correlated with ferroptosis (*Acsl4*, *Hmox1*, *Fth1*, *Cybb*, *Lcn2*, *slc7a11*, *Ptgs2*), osteoclast differentiation (*Tnfsf11*, *Csf1*), toll‐like receptor (TLR) signaling (*Myd88*, *Irf7*, *Fos*, *Ikbke*), necroptosis and apoptosis were upregulated in *E. coli*‐infected and *S. aureus*‐infected BMSCs. Part of DEGs related to ferroptosis were validated by qRT‐PCR as shown in Figure S3B (Supporting Information).

The results of the KEGG pathway enrichment analysis (Figure 3D,E) demonstrated that ferroptosis displayed the highest enrichment factor among all death‐related signaling pathways in either *E. coli*‐infected and *S. aureus*‐infected BMSCs, which is consistent with the former results in vitro and in vivo and consolidated the predominant role of ferroptosis in bacteria‐induced death. In addition, our analysis also spotlighted significant alterations in the toll‐like receptor signaling pathway in BMSCs infected by either bacterial strain. The significant change in the TLR signaling pathway was subsequently confirmed through the GSEA enrichment analysis, as visualized in Figure 3H. These results consolidated the occurrence of ferroptosis and osteogenic dysfunction in *S. aureus*‐infected and *E. coli*‐infected BMSCs at the molecular level and unveiled the activation of innate immune responses of infected BMSCs.

### 2.4 TLR Signaling and IRF7 Regulate Infection‐Induced BMSCs Ferroptosis

**Figure 4:**
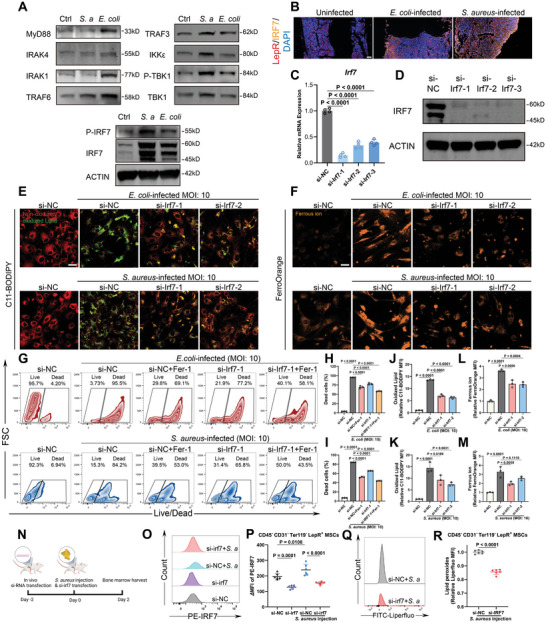
Toll‐like receptor signaling and IRF7 regulate infection‐induced BMSC ferroptosis. A) The regulating effects of *S. aureus* and *E. coli* infections on toll‐like receptor signaling proteins including MyD88, IRAK4, IRAK1, TRAF6, TRAF3, IKKε, p‐TBK1/TBK1, and p‐IRF7/IRF7. B) Representative tissue immunofluorescence images of LepR, IRF7 in the uninfected murine femur and implant‐associated bone infection model induced by *S. aureus* and *E. coli*. Scale bar = 50 µm. C) IRF7 knockdown efficiency was evaluated by qRT‐PCR. The *Hsp90b1* gene was used as an internal reference gene. Values are means ± SDs (*n* = 4). Multiple comparison was performed by one‐way analysis of variance (ANOVA) with Tukey's post‐hoc analysis. D) WB analysis of IRF7 knockdown efficiency. E,F) Representative fluorescent images of C11‐BODIPY staining (Scale bar = 40 µm) and FerroOrange staining (Scale bar = 40 µm). BMSCs were transfected with indicated siRNA for 48 h and then infected with *S. aureus* (MOI: 10) and *E. coli* (MOI: 10) for 12 h before observation. G) The effects of IRF7 on *S. aureus* and *E. coli*‐induced BMSC death were analyzed by IRF7 knockdown and Live/Dead staining assay and flow cytometry analysis. BMSCs were transfected with indicated siRNA for 48 h and then infected with *S. aureus* (MOI: 10) and *E. coli* (MOI: 10) for 12 h before analysis. H,I) Quantification of the rate of dead cells in flow cytometry analysis in (G). Values are means ± SDs (*n* = 3). Multiple comparison was performed by one‐way analysis of variance (ANOVA) with Tukey's post‐hoc analysis. Quantification of fluorescent intensity of oxidized lipid J,K), ferrous iron L,M). Values are means ± SDs (*n* = 3). Multiple comparison was performed by one‐way analysis of variance (ANOVA) with Tukey's post‐hoc analysis. N) The schematic route of animal experiments of in vivo si‐RNA transfection in murine osteomyelitis model. Each group contained six mice. O) Representative flow cytometry images of PE‐IRF7 in CD45^−^CD31^−^Ter119^−^LepR^+^ BMSCs in mice transfected with si‐RNA. P) Quantitative analysis of the median fluorescent intensity of PE‐IRF7 in (O), Values are means ± SDs (*n* = 6). Multiple comparison was performed by one‐way analysis of variance (ANOVA) with Tukey's post‐hoc analysis. Q) Representative flow cytometry images of lipid peroxides stained by Liperfluo in CD45^−^CD31^−^Ter119^−^LepR^+^ BMSCs were analyzed in a murine osteomyelitis model transfected with si‐RNA. R) Quantitative analysis of the median fluorescent intensity of Lipid peroxides in (O), Values are means ± SDs (*n* = 6). Comparison between two groups was performed by a two‐tailed Student's *t‐test*.

Mounting evidence supported a strong connection between innate immune response and ferroptosis.^[^ ^17^ ^]^ TLR and their downstream signaling take a vital role in recognizing pathogen invasion and initiating antibacterial inflammation responses.^[^ ^18^ ^]^ In the results of RNA‐Seq, the toll‐like receptor signaling pathway was significantly altered in both *S. aureus*‐infected and *E. coli*‐infected BMSCs. In addition, interferon regulatory factor 7 (IRF7) was the highest upregulated transcriptional factor in BMSCs either infected with *S. aureus* or *E. coli* (Figure 3I). This evidence leads us to the hypothesis postulating a potential interlinkage between the activation of the TLR signaling pathway and the onset of ferroptosis. Expression levels of downstream signaling molecules of toll‐like receptor signaling were determined by western blot. As shown in **Figure** **4**A, *E. coli*‐infection induced upregulation of IRF7 and phosphorylation of IRF7 via activating MyD88‐IRAK4‐IRAK1/TRAF6 axis. Different from *E. coli* infection, *S. aureus* infection induced IRF7 and phosphorylation of IRF7 through activating TRAF3‐IKKε/TBK1 axis, bypassing MyD88. This differential modulation by the two bacterial strains may be attributed to their unique molecular patterns interacting with specific TLR on BMSCs. Subsequent in vivo validation was performed by evaluating IRF7 expression in LepR^+^ cells within an implant‐associated bone infection model. The immunofluorescence staining reinforced our in vitro observations, demonstrating a pronounced upregulation of IRF7 (Figure 4B).

To precisely delineate the role of IRF7 in mediating bacteria‐induced ferroptosis in BMSCs, si‐RNAs targeting IRF7 were designed to knock down IRF7. As evidenced in Figure 4C,D, all three si‐RNAs targeting Irf7 (namely, si‐Irf7‐1, si‐Irf7‐2, and si‐Irf7‐3) proficiently reduced the expression of IRF7 both at the mRNA and protein levels. Then si‐Irf7‐1 and si‐Irf7‐2 were selected for the following experiments.

In the bacteria‐BMSCs co‐culture model, as shown in Figure 4E,F, IRF7 silence in BMSCs significantly alleviated the upsurge of lipid peroxides and ferrous ions triggered by *S. aureus* and *E. coli* infections. Quantitative analysis of fluorescence intensity consolidated these findings (Figure 4J–M). Based on these results, the effects of Irf7 knockdown or combined with Fer‐1 on bacteria‐triggered ferroptosis were investigated with Live/Dead flow cytometry analysis. The results (Figure 4G–I) demonstrated that knockdown of IRF7 mitigated the death rate of BMSCs infected by *S. aureus* and *E. coli*, and a combined regimen of Fer‐1 and si‐Irf7 manifested pronounced synergistic anti‐ferroptosis effects in the context of both bacterial infections.

To validate the modulatory influence of IRF7 on ferroptosis in vivo, IRF7 was silenced through intratibial injections of si‐Irf7 in a murine osteomyelitis model induced by *S. aureus* infection (Figure 4N). Figure 4O,P showed that in vivo intratibial si‐RNA transfection effectively downregulated the expression of IRF7 in CD45^−^CD31^−^Ter119^−^LepR^+^ BMSCs in uninfected mice or osteomyelitis mice with *S. aureus* injection. Subsequent results confirmed that IRF7 knockdown alleviated lipid peroxide levels in CD45^−^CD31^−^Ter119^−^LepR^+^ BMSCs in the bone marrow, as depicted demonstrated in Figure 4Q,R.

### 2.5 Infection‐Induced Upregulation of ACSL4 was Transcriptionally Activated by IRF7

**Figure 5:**
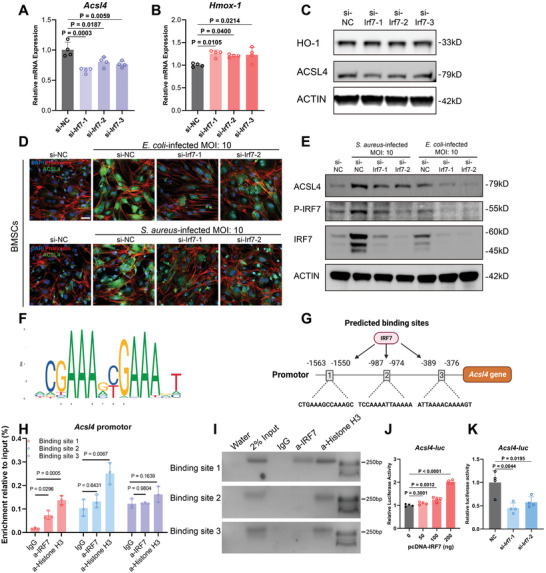
Infection‐induced upregulation of ACSL4 was transcriptionally activated by IRF7. A–C) Regulating effects of IRF7 silencing on *Acsl4* and *Hmox‐1* expression at mRNA level and protein level. For qRT‐PCR, Values are means ± SDs (*n* = 4). Multiple comparison was performed by one‐way analysis of variance (ANOVA) with Tukey's post‐hoc analysis. D) Representative images of cell immunofluorescence of BMSCs stained with ACSL4 (green), Phalloidin (red) and DAPI (blue). BMSCs were transfected with indicated siRNA for 48 h and then infected with *S. aureus* (MOI: 10) and *E. coli* (MOI: 10) for 12 h before observation. Scale bar = 40 µm. E) The regulation effects of IRF7 silencing on bacterial infection‐induced ACSL4 upregulation were analyzed by WB analysis. F) Illustration of the binding motif of IRF7. G) Schematic diagrams of potential IRF7 binding site on Acsl4 promoter covering 2‐kb nucleotide sequences upstream from the transcription initiation site. Binding site prediction was performed using the JASPAR database. H) Promoter occupancy analysis of IRF7 in Acsl4 promoter using ChIP‐PCR assay in BMSCs cells. Values are means ± SDs (*n* = 3). Multiple comparison was performed by one‐way analysis of variance (ANOVA) with Tukey's post‐hoc analysis. I) Representative images of agarose gel electrophoresis of ChIP‐PCR assay. The regulating effects of IRF7‐overexpression J) and IRF7 knockdown K) on Acsl4 promotor activity were determined by dual‐luciferase reporter assay. Values are means ± SDs (*n* = 4). Multiple comparison was performed by one‐way analysis of variance (ANOVA) with Tukey's post‐hoc analysis.

To elucidate the mechanistic underpinnings by which IRF7 modulates ferroptosis, we executed an IRF7 knockdown in BMSCs and subsequently probed its regulatory impact on the expression of ferroptosis‐associated genes, as identified through RNA‐seq and qRT‐PCR analysis. **Figure** **5**A–C distinctly illustrated that the suppression of IRF7 leads to a marked downregulation of ACSL4 both at mRNA and protein levels. IRF7 knockdown moderately upregulated mRNA expression of *Hmox1*, but did not have an observable effect on HO‐1 expression at the protein level. Furthermore, both immunofluorescence and western blot assays underscored that IRF7 silencing mitigated the upregulated expression of ACSL4 in BMSCs infected with *S. aureus* and *E. coli* (Figure 5D,E). Functionally, ACSL4 catalyzes the synthesis reaction of polyunsaturated fatty acids‐containing phospholipids (PUFA‐PLs) and directly contributes to the occurrence of ferroptosis.^[^ ^12^, ^19^ ^]^ Ferroptosis could be blocked by inactivation or knockout of ACSL4.^[^ ^20^ ^]^ Collectively, these findings substantiate the pivotal role of IRF7 in modulating ACSL4 expression, thereby shedding light on the potential mechanism through which IRF7 exerts control over ferroptosis. Given the canonical role of IRF7 as a transcription factor, primarily influencing biological outcomes by modulating downstream gene expression, it is plausible to surmise that its regulatory influence on ACSL4 operates through transcriptional modulation.

To validate our hypothesis that IRF7 directly regulates ACSL4 transcription, transcription factor binding site predictions, chromatin immunoprecipitation (ChIP) and dual‐luciferase reporter gene assay were performed. First, we used the JASPAR database to analyze potential IRF7 binding sites within the 2000 bp upstream promoter sequence of the *Acsl4* gene. Based on the binding motif of IRF7 (Figure 5F), the computational analysis (Figure 5G) demonstrated that there existed three potential binding sites, from −1563 to −1550 bp (Binding site 1), from −987 to −974 bp (Binding site 2), and from −389 to −376 bp (Binding site 3). ChIP‐PCR assay in BMSCs was performed with IRF7 antibody to verify whether IRF7 could directly bind to these sites. The results of ChIP‐PCR (Figure 5H,I) showed that IRF7 bound only to binding site 1. Moreover, to validate the regulating effects of IRF7 on *Acsl4* promotor activity, the *Acsl4* promotor fragment was fused to a luciferase reporter plasmid (pGL4.10). And dual luciferase reporter assay was performed in BMSCs. The results (Figure 5J,K) indicated that overexpression of IRF7 with pcDNA‐IRF7 plasmid increased *Acsl4* promotor activity in a dose‐dependent manner, while silencing IRF7 by siRNA knockdown significantly inhibited *Acsl4* promoter activity. In summary, these results demonstrated that IRF7 acts as a transcription stimulator of *Acsl4* in BMSCs, which further contributed to bacteria‐induced ferroptosis in BMSCs.

### 2.6 Targeting‐Ferroptosis Rescued Osteogenic Differentiation Potential of Infected BMSCs

**Figure 6:**
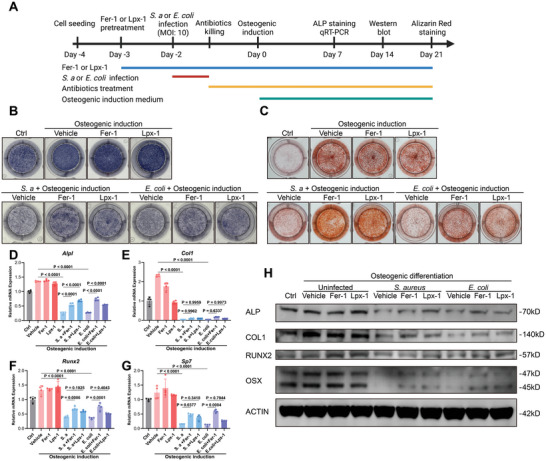
Targeting ferroptosis rescued osteogenic differentiation potential of infected BMSCs. A) Schematic diagram of osteogenic differentiation of BMSCs after *S. aureus* and *E. coli* infections. The single rescuing effects of ferroptosis inhibitors Fer‐1 (10 µm) or Lpx‐1 (1 µm) on osteogenic differentiation of infected BMSCs were measured by ALP staining, Alizarin Red S staining, qRT‐PCR and WB at the indicated time. B) Representative images of ALP staining on Day 7 post osteogenic induction. C) Representative images of Alizarin Red S staining on Day 21 post osteogenic induction. D–G) Gene expression of *Alpl*, *Col1*, *Runx2*, *Sp7* was determined by qRT‐PCR on Day 7 post osteogenic induction. The *Hsp90b1* gene was used as an internal reference gene. Values are means ± SDs (*n* = 4). Multiple comparison was performed by one‐way analysis of variance (ANOVA) with Tukey's post‐hoc analysis. H) WB analysis of the expression of ALP, COL1, RUNX2, OSX in BMSCs on Day 14 post osteogenic induction.

Our preliminary investigations highlighted the dominant role of ferroptosis in the damage and subsequent bacteria‐induced mortality of BMSCs. Based on these findings, we hypothesized that obstructing ferroptosis with specific inhibitors could rescue cell death and restore osteogenic differentiation potential in BMSCs. To validate this hypothesis, we designed a consecutive experiment involving ferroptosis inhibition and osteogenic induction in a bacteria‐BMSCs co‐culture model (**Figure** **6**A).

We first evaluate osteogenic differentiation by Alkaline Phosphatase (ALP) staining and Alizarin Red S (ARS) staining on Day 7 and Day 21 post‐osteogenic induction, respectively. Interestingly, the findings (Figure 6B,C) revealed that the application of Fer‐1 (10 µm) and Lpx‐1 (1 µm) to uninfected BMSCs had no discernible impact on standard osteogenic differentiation when compared with the vehicle group. However, a significant inhibitive effect in osteogenic differentiation emerged in BMSCs exposed to *S. aureus* (MOI: 10) and *E. coli* (MOI: 10). Fer‐1 and Lpx‐1 notably enhanced ALP levels and calcified node formation relative to their respective vehicle cohorts (*S. aureus* and *E. coli* post‐infection).

This restorative effect of ferroptosis inhibitors on osteogenic capability was further corroborated by qRT‐PCR and western blot analysis. As delineated in Figure 6D–G, the expression profiles of key osteogenic markers in BMSCs on Day 7—namely *Alpl*, *Col1*, *Runx2*, and *Sp7*—were markedly suppressed upon *S. aureus* and *E. coli* exposure. Strikingly, this suppression was effectively alleviated by the ferroptosis inhibitors Fer‐1 and Lpx‐1. Expression of these genes on Day 14 at the protein level also showed a similar trend (Figure 6H).

Collectively, our results underscore the potential of Fer‐1 and Lpx‐1 in ameliorating the osteogenic dysfunction in BMSCs infected by *S. aureus* and *E. coli* infections. These findings suggest that ferroptosis‐targeted intervention may serve as a promising therapeutic strategy for the regeneration of infected bone defects.

### 2.7 Synthesis of ROS‐Responsive Dual‐Functional Antimicrobial/Anti‐Ferroptosis 3D‐Printed Scaffold

**Figure 7:**
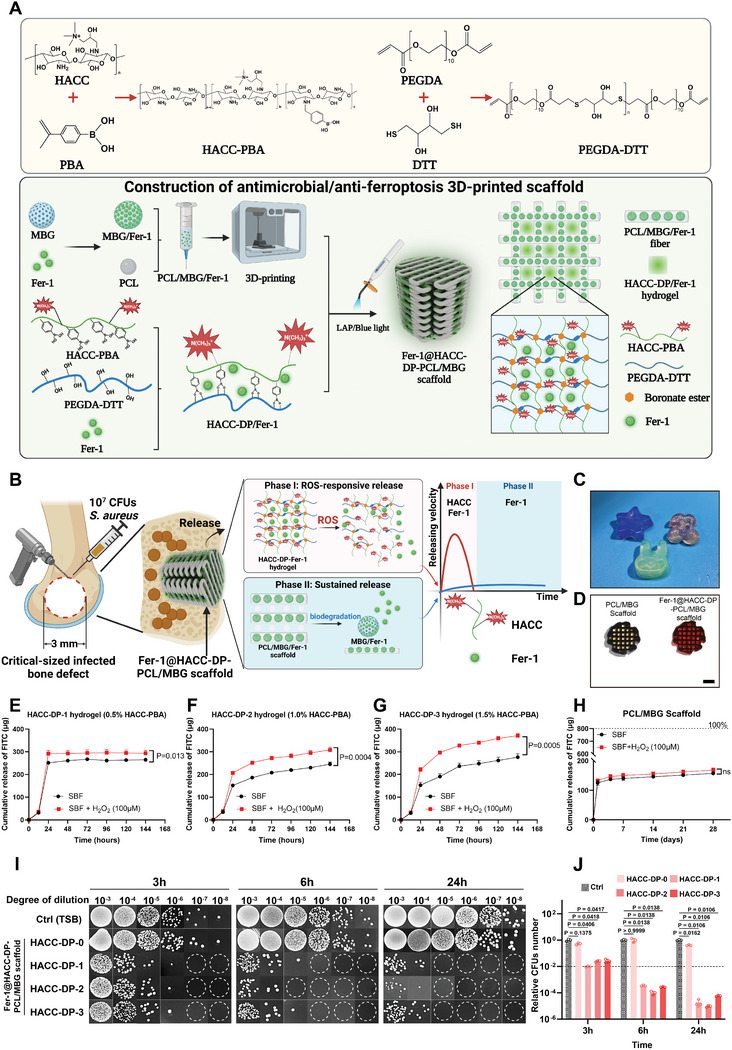
Synthesis of ROS‐responsive HACC‐DP/Fer‐1 hydrogel and construction of antibacterial/anti‐ferroptosis 3D printed scaffold. A) Schematic illustration showing synthesis route of ROS‐responsive HACC‐DP/Fer‐1 hydrogel and construction of antibacterial/anti‐ferroptosis 3D printed scaffold. B) Schematic illustration showing surgical procedures of animal model and two‐phase release of HACC and Fer‐1 from the antibacterial/anti‐ferroptosis 3D printed scaffold. C) Representative images of shape adaptability of HACC‐DP/Fer‐1 hydrogel containing 1% HACC‐PBA in different patterns. Detailed contour indicates flexible in‐situ shape adaptability. D) Images of PCL/MBG scaffold and Fer‐1@HACC‐DP‐PCL/MBG scaffold (Scale bar = 1 mm). Cumulative release FITC in HACC‐DP/FITC hydrogel containing 0.5% HACC‐PBA E), 1.0% HACC‐PBA F), 1.5% HACC‐PBA G) and PCL/MBG/FITC scaffold H) in SBF and SBF+H_2_O_2_ (100 µm) at 37 °C were measured at predetermined time points. A significantly higher release pattern in SBF+H_2_O_2_ compared with SBF indicated the ROS‐responsive release capability of HACC‐DP hydrogel. Values are means ± SDs (*n* = 3). Two‐way analysis of variance (ANOVA) was performed. I) Representative images of CFUs counting in *S. aureus* treated by Fer‐1@HACC‐DP‐PCL/MBG scaffolds with different HACC‐PBA content for 3, 6, and 24 h. J) Quantitative analysis of CFU number in (I). Values are means ± SDs (*n* = 3). Multiple comparison was performed by one‐way analysis of variance (ANOVA) with Tukey's post‐hoc analysis.

Ideal bone repair materials for infected bone defects are proposed to release antimicrobial and tissue‐protecting factors in an infection‐responsive manner in the early stage of bone infection, which could adapt to the dynamic pathogen burden and inflammation load. In the middle and late stages of bone repair, sustained release of tissue‐protecting factors is required to maintain a homeostatic repair environment. Based on this concept, we sought to develop a bone repair scaffold that integrates a ROS‐responsive hydrogel with a 3D‐printed scaffold, offering adjustable release of antimicrobial and anti‐ferroptosis agents in early stage and sustained release of anti‐ferroptosis agents in the middle and late stage of bone repair (**Figure** **7**B).

A notable characteristic of infected sites is the heightened production of ROS due to inflammation,^[^ ^21^ ^]^ which can be harnessed for controlled release. Our prior research spotlighted the effectiveness of a particular quaternized chitosan (hydroxypropyltrimethyl ammonium chloride chitosan, HACC) with a 26% substitution degree. It showed notable antimicrobial activity while simultaneously facilitating bone regeneration.^[^ ^22^ ^]^ This led to our proposal of synthesizing a photo‐cross‐linking hydrogel that would exhibit ROS‐responsive release dynamics of both HACC and Fer‐1.

Illustrated in Figure 7A, we modified HACC with phenylboronic acid, obtaining HACC‐PBA. In a parallel process, Poly (ethylene glycol) diacrylate (PEGDA) and DL‐Dithiothreitol (DTT) were combined to create a photo‐cross‐linking polymer named PEGDA‐DTT, which could form ROS‐responsive boronic esters in alliance with the PBA group from HACC‐PBA. Next, biodegradable scaffolds, formulated from a blend of polycaprolactone, and mesoporous bioactive glass loaded with Fer‐1, were constructed using 3D printing. This scaffold was designed to provide sustained tissue protection and osteoinduction. Before inducing photo‐cross‐linking within the 3D‐printed scaffold, we added a calculated quantity of HACC‐PBA and Fer‐1 into the PEGDA‐DTT solution, and stirred for 2 h, yielding HACC‐DP/Fer‐1. Subsequently, this mixture, complemented with the photo‐initiator Lithium Phenyl (2,4,6‐trimethyl benzoyl) phosphinate (LAP), was added into the PCL/MBG/Fer‐1 scaffold. In‐situ photo‐cross‐linking was triggered by blue light exposure, yielding the composite Fer‐1@HACC‐DP‐PCL/MBG scaffold.

HACC‐PBA, the pivotal component of the HACC‐DP hydrogel, was synthesized successfully, as evidenced in Figure S4A,B (Supporting Information). The synthesis was authenticated by both Fourier‐transform infrared spectroscopy (FTIR) and ^1^H‐nuclear magnetic resonance (^1^H NMR). In the FTIR spectrum, a characteristic peak at 1344 cm^−1^ corresponding to B‐O was observed. Similarly, the ^1^H NMR spectrum displayed characteristic proton signals: one at 7.11 ppm (‐BOH) and another at 7.47 ppm (‐C6H4‐).^[^ ^23^ ^]^ Based on these findings, we initiated the photo‐cross‐linking of the HACC‐DP hydrogel. Observations from Figure S4D (Supporting Information) indicated that the hydrogel efficiently formed under blue light irradiation when the HACC‐PBA content ranged between 0% and 1.5%. The formation of HACC‐DP hydrogel was further validated by a rheological test (Figure S4E, Supporting Information), which demonstrated that the storage modulus (G’) value was higher than the loss modulus (G’’) value in all HACC‐DP hydrogel groups after blue light irradiation. The results (Figure S4G, Supporting Information) of shear‐thinning effect and injectability tests indicated that HACC‐DP hydrogels had relative good injectability and suitable for filling in scaffolds. The shape adaptability of the HACC/DP hydrogels, depicted in Figure 7C, emphasizes its compatibility with the geometry of the PCL/MBG scaffold. As illustrated in Figure 7D, both the PCL/MBG/Fer‐1 scaffold and the integrated Fer‐1@HACC‐DP‐PCL/MBG scaffold were effectively constructed. The compression modulus (Figure S4C, Supporting Information) of both scaffolds reached over 20 MPa, which were relative higher in 3D printed scaffold and suitable for bone repair.

### 2.8 In Vitro and In Vivo Degradation Test and Evaluation of Antibacterial Effects

We sought to investigate the release behavior of the Fer‐1@HACC‐DP‐PCL/MBG scaffold. For this, fluorescein isothiocyanate (FITC), a small fluorescent molecule, was employed to simulate the Fer‐1 release in both HACC‐DP/Fer‐1 hydrogel and PCL/MBG/Fer‐1 scaffold within simulated body fluid (SBF) in vitro. In our observations (Figure 7E–G), HACC‐DP/FITC hydrogel with HACC‐PBA concentrations of 1% and 1.5% demonstrated a consistent FITC release over 6 days in SBF. In contrast, hydrogel containing 0.5% HACC‐PBA almost exhibited complete release within the initial 24 h. Interestingly, when the SBF was supplemented with 100 µm H_2_O_2_, FITC release from the HACC‐DP/FITC hydrogel was notably accelerated, underlining the hydrogel's distinct ROS‐responsive release capabilities. In the case of the PCL/MBG/FITC scaffold (Figure 7H), we noted an initial burst of FITC release within the first 24 h, which then transitioned into a more controlled and steady release phase. Different from ROS‐responsive hydrogel, the presence or absence of H_2_O_2_ in the SBF did not manifest in significant differences in FITC release patterns. By the end of the observation period at 28 days, only ≈19.5% of the total FITC content (equivalent to 800 µg) in the scaffold had been released, aligning with the scaffold's gradual biodegradation, as further illustrated in Figure S4I–L (Supporting Information).

The antimicrobial efficacy of the Fer‐1@HACC‐DP‐PCL/MBG scaffold containing different HACC‐DP hydrogel was subsequently evaluated. As shown in Figure 7I,J, the scaffolds with HACC‐DP‐1, HACC‐DP‐2 and HACC‐DP‐3 hydrogels exhibited an inhibition rate of above 90% against planktonic *S. aureus* in tryptic soy broth (TSB) at 3 h. The bacteria level decreased by four orders of magnitude at 24 h compared with those in Ctrl group and scaffold with HACC‐DP‐0. This result demonstrated that the scaffold with HACC‐DP‐1, HACC‐DP‐2 and HACC‐DP‐3 hydrogels could effectively inhibit the growth of planktonic bacteria. The scanning electronic microscopy (SEM) was used to observe the structure change of *S. aureus* and biofilm formation on scaffolds in this study. As shown in Figure S4H (Supporting Information), Bacteria on PCL/MBG scaffold and Fer‐1@HACC‐DP‐0‐PCL/MBG scaffold (without HACC‐PBA in hydrogel) showed intact round shape and mature biofilm on scaffolds. In contrast, no biofilm was observed on Fer‐1@HACC‐DP‐2‐PCL/MBG scaffold (containing 1.0% HACC‐PBA in hydrogel), with few dead bacteria with pore formed on bacteria surface. This indicated that bacterial cell wall was damaged by HACC‐DP‐2 hydrogel. We also evaluated the biocompatibility of the Fer‐1@HACC‐DP‐PCL/MBG scaffold. As illustrated in Figure S4M (Supporting Information), BMSCs could proliferate on Fer‐1@HACC‐DP‐PCL/MBG scaffolds and cell viability of BMSCs grown on Fer‐1@HACC‐DP‐PCL/MBG scaffold was above 80% compared with BMSCs seeded in culture disk (control), which showed good biocompatibility of Fer‐1@HACC‐DP‐PCL/MBG scaffolds. Given these findings, we opted to utilize the HACC‐DP hydrogel with 1% HACC‐PBA for the subsequent fabrication of the composite scaffold and the ensuing in vivo evaluations.

To clarify the relationship of in vivo degradation of hydrogel and antibacterial effects, we conducted a subcutaneous degradation test of HACC‐DP‐2/Fer‐1 hydrogel. As indicated in Figure S5A (Supporting Information), the results of H&E staining demonstrated that the HACC‐DP‐2/Fer‐1 hydrogel (indicated by blue arrow) implanted in the uninfected subcutaneous cavity gradually degraded from Day 1 to Day 7 post‐operation and fully degraded by Day 14 post‐operation. Correspondingly, the subcutaneous cavity gradually closed, and healing tissue formed on Day 14. In contrast, the degradation rate of HACC‐DP‐2/Fer‐1 hydrogel was markedly accelerated by subcutaneous infection microenvironment. The HACC‐DP‐0/Fer‐1 hydrogel followed a similar degradation pattern as the HACC‐DP‐2/Fer‐1 hydrogel.

To clarify the antibacterial effects of these hydrogels, we conducted Giemsa staining to label residual bacterial in tissue. As shown in Figure S5B (Supporting Information), bacteria could be observed in subcutaneous tissue around the implanted hydrogels in HACC‐DP‐2/Fer‐1 and HACC‐DP‐0/Fer‐1 with infection on Day 1. As the release and degradation of HACC‐DP‐2/Fer‐1 hydrogel occurred, bacterial colonies were undetectable from Day 3 to Day 14. However, dense bacterial colonies could be observed in tissue in HACC‐DP‐0/Fer‐1 group from Day 3 to Day 14. This result demonstrated that the release and degradation of HACC‐DP‐2/Fer‐1 hydrogel could exert effective antibacterial effects on subcutaneous *S. aureus* compared with HACC‐DP‐0 hydrogel. The antibacterial effects could also be reflected by the level of tissue‐infiltrated inflammatory cells from histological evaluation. As shown in high magnification images in H&E staining (Figure S5A, Supporting Information), dense inflammatory cell infiltration could be observed in tissue around hydrogel in HACC‐DP‐0/Fer‐1 hydrogel group from Day 1 to Day 7, and abscess tissue formed on Day 14, indicating persistent bacterial activity. In contrast, the level of inflammatory cell infiltration in HACC‐DP‐2/Fer‐1 group was moderate compared to that in HACC‐DP‐0/Fer‐1 group from Day 1 to Day 7 and healing tissue successfully formed on Day 14, indicating effective elimination of tissue bacteria.

The overall results demonstrated that the HACC‐DP‐2/Fer‐1 hydrogel could degraded in an infection‐responsive pattern from Day 1 to Day 7 in vivo and exert potent antibacterial effect on *S. aureus* infection, leading to lower tissue inflammatory level and tissue healing.

### 2.9 Intervention in Ferroptosis Facilitates Osteogenic Regeneration of Infected Bone Defect in a Rat Model

**Figure 8:**
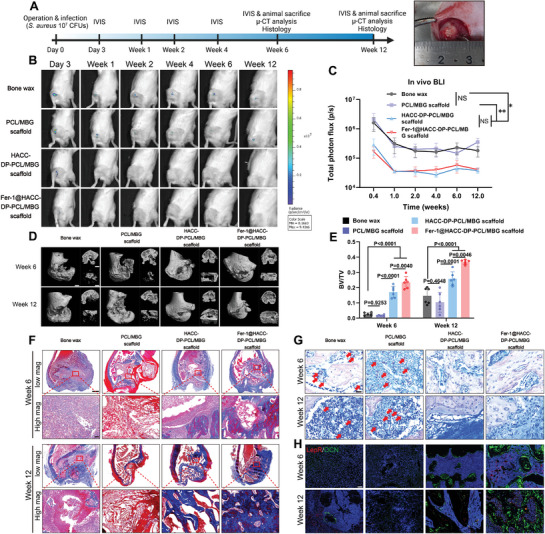
Intervention in ferroptosis facilitates regeneration of infected bone defect in a rat infected distal‐femur defect model. A) Schematic diagram of animal experiment. A critical‐sized bone defect with a diameter of 3 mm and a depth of 4 mm was made on the distal femur of rats. 50 µL of 2 × 10^8^ CFUs mL^−1^ *S. aureus* (*Xen 29*) suspension was injected into the defect sites. Bone wax or different scaffolds were then implanted into defect sites. Each group contained 12 rats, with 6 rats sacrificed at Week 6 and 6 rats sacrificed at Week 12 post‐operation. B) Representative of in vivo bioluminescence images from rats in each group. Bioluminescence reflected bacterial burden at the operation sites. C) Quantitative analysis of total photon flux at operation sites. Values are means ± SDs (*n* = 12 for all groups from Day 3 to Week 6, *n* = 6 for all groups at Week 12). Multiple comparison was performed by two‐way analysis of variance (ANOVA) with Tukey's post‐hoc analysis. * indicates *P* < 0.05, ** indicates *P* < 0.01. D) Representative of micro‐CT images from rats in each group at Week 6 and Week 12. The overall 3D reconstruction view was on the left (Scale bar = 1 mm). The coronal plane view was on the top right corner (Scale bar = 0.5 mm). The 3D reconstruction image of bone regeneration at defect sites was on the bottom right corner (Scale bar = 0.5 mm). E) Quantitative analysis of BV/TV at defect sites. Values are means ± SDs (*n* = 6). Multiple comparison was performed by one‐way analysis of variance (ANOVA) with Tukey's post‐hoc analysis. F) Representative images of Masson trichrome staining from rats in each group at Week 6 and Week 12. Scale bar = 1 mm (low magnification). Scale bar = 50 µm (high magnification). G) Representative images of Giemsa staining from rats in each group at Week 6 and Week 12. Scale bar = 25 µm. H). Representative immune fluorescence images of OCN (Green) and LepR (Red) from rats in each group at Week 6 and Week 12. Scale bar = 100 µm.

To assess the therapeutic effectiveness of the antimicrobial/anti‐ferroptosis scaffold in the repair of infected bone defects, we created critical‐sized infected bone defect models in the distal femur of rats. The study comprised four groups based on the implanted materials: Bone wax (blank control), PCL/MBG scaffold (lacking antimicrobial/anti‐ferroptosis functions), HACC‐DP‐PCL/MBG scaffold (antimicrobial properties but without anti‐ferroptosis features), and Fer‐1@HACC‐DP‐PCL/MBG scaffold (possessing both antimicrobial and anti‐ferroptosis characteristics). The procedural timeline is illustrated in **Figure** **8**A.

The in vivo imaging system (IVIS) was utilized for continuous monitoring of bacterial loads. Figure 8B,C displayed those bacterial signals persisted in the surgical sites of the Bone wax and PCL/MBG scaffold groups, detectable from Day 3 up to Week 12. In contrast, the groups with HACC‐DP‐PCL/MBG and Fer‐1@HACC‐DP‐PCL/MBG scaffold exhibited a marked reduction in bacterial presence on Day 3 post operation, with undetectable levels from Week 1 to Week 12. This suggested that the HACC‐DP hydrogel within the scaffold effectively eradicated bacteria at the defect sites.

At the predetermined time points of Week 6 and Week 12 post‐surgery, rats were sacrificed, and their femurs were analyzed using micro‐CT, X‐ray and histological evaluations. The micro‐CT results (Figure 8D) revealed significant osteolysis and pathological fractures in the Bone wax and PCL/MBG scaffold groups on Week 6. By 12 weeks post‐operation, the pathology deteriorated further, with substantial fractures, pronounced joint damage, and deformities. In contrast, bone regeneration and reactive new bone formation were observed in the defect periphery in groups with HACC‐DP‐PCL/MBG scaffold and Fer‐1@HACC‐DP‐PCL/MBG scaffold on Week 6. The central defect region exhibited signs of bone formation, especially evident in the Fer‐1@HACC‐DP‐PCL/MBG scaffold group on Week 12. Quantitative micro‐CT analysis (Figure 8E) revealed that new bone formation (BV/TV) in the group with Fer‐1@HACC‐DP‐PCL/MBG scaffold surpassed those in the group with HACC‐DP‐PCL/MBG at both the 6 and 12‐week time points. Furthermore, BV/TV in the Fer‐1@HACC‐DP‐PCL/MBG and HACC‐DP‐PCL/MBG scaffold groups significantly exceeded those in the Bone wax and PCL/MBG scaffold groups. The X‐ray images (Figure S6, Supporting Information) were also consistent with the results of micro‐CT. Conclusively, while scaffolds possessing only antimicrobial function moderately prevent osteolysis, joint destruction, and support bone regeneration in critical‐sized infected bone defects, targeting ferroptosis to mitigate cell damage substantially augments bone repair in these defects.

We further carried out histological analysis to evaluate the therapeutic efficacy of these scaffolds. As shown in Masson trichrome staining (Figure 8F), a large amount of granular tissue and fibrous tissue filled the defect in the bone wax group on Week 6 and Week 12, which showed that the critical‐sized infected bone defect could not be repaired by self‐healing. In the PCL/MBG scaffold group on Week 6, an apparent fibrous capsule formed around the scaffold and fibrous tissue filled the defect. This situation worsened on Week 12, with the scaffold being completely squeezed out of the defect area by fibrous tissue. Meanwhile, in the HACC‐DP‐PCL/MBG group, the new bone tissue could be observed around the scaffold on Week 6 and more appeared on Week 12. Fer‐1@HACC‐DP‐PCL/MBG group showed the most apparent trabecular bone tissue in the central defect area on Week 6 and Week 12, which validates the results of micro‐CT analysis. The Giemsa staining (Figure 8G) indicates the bacteria presence in the groups with bone wax and PCL/MBG scaffold, but not in the groups with HACC‐DP‐PCL/MBG scaffold and Fer‐1@HACC‐DP‐PCL/MBG scaffold, which demonstrated that HACC containing scaffolds could effectively eradicate pathogen in infected bone defects. The tissue immunofluorescence (Figure 8H) showed that LepR and osteocalcin (OCN) signals in the HACC‐DP‐PCL/MBG scaffold were significantly higher than other three groups, which reflected more BMSCs and more mature osteogenic differentiation induced by Fer‐1 releasing scaffold.

In summary, the above results demonstrate that ROS‐responsive releasing of HACC and sustained delivery of Fer‐1 could effectively eradicate bacteria and protect the cell viability of BMSCs, thus promoting bone regeneration in infected bone defect models.

## 3 Discussion

During the pathological progression of infected bone defects, the persistence of viable bacterial pathogens and their associated metabolites induce reprogramming of cells within the infection microenvironment. While several virulence factors from *S. aureus* and *E. coli* have been identified for their direct cytotoxicity,^[^ ^8^, ^24^ ^]^ the direct regulatory impacts of living bacteria and their metabolites on bone progenitor cells remain elusive. The mechanisms by which living bacteria induce cell death may diverge from those induced by individual bacterial components. Numerous studies support the notion of intracellular *S. aureus* behaving as a pathogen within osteoblasts, osteocytes, and macrophages, further complexifying the cellular injury and death mechanisms.^[^ ^10^, ^25^ ^]^ In this study, we identified the predominant cell death pathways in BMSCs upon infection with *S. aureus* and *E. coli*, using an array of cell death inhibitors. Our findings demonstrated a pronounced ferroptosis phenotype in infected BMSCs, characterized by increased intracellular lipid peroxides, overload of ferrous iron, distinctive mitochondrial morphology, and the upregulation of ferroptosis‐related genes. In vivo evidence, including the heightened protein modifications of lipid peroxidation products in the infected bone marrow and amplified lipid peroxides in CD45^−^CD31^−^Ter119^−^LepR^+^ BMSCs, corroborated the manifestation of ferroptosis in a bone infection environment.

Recently, the role of ferroptosis in bacterial‐induced cell death has been thrust into the spotlight. For instance, both *M. tuberculosis* and *P. aeruginosa* have been reported to induce ferroptosis within macrophages and bronchial epithelium cells, respectively.^[^ ^13a,c^ ^]^ Certain bacterial components, such as Tyrosine phosphatase A (PtpA) and lipoxygenase (pLoxA), have been pinpointed as ferroptosis triggers in infections by these bacteria. The virulence factors of *S. aureus* and *E. coli* involved in inducing ferroptosis remain to be explored and elucidated. We also observed significant alterations in the expression of genes related to apoptosis, pyroptosis, and necroptosis pathways, as evidenced by the elevated expression levels of *Casp7*, *Casp1*, *Nlrp3*, and *Mlkl*. This suggests that a multitude of death signaling pathways might converge to determine the injury mechanisms in bacteria‐induced BMSC death. Emerging evidence indicates potential interactions between different death signaling pathways. For instance, Caspase 7 (Casp7) has been noted to activate acid sphingomyelinase (ASM) to mend gasdermin and perforin pores in infected hepatocytes.^[^ ^26^ ^]^ This reparative mechanism potentially defers pore‐driven lysis, steering the cell toward a moderated death program. In light of this, it is plausible that multiple death signaling pathways in BMSCs are co‐activated upon infection with *S. aureus* and *E. coli*. These pathways might interplay and culminate in a predominant ferroptosis death phenotype.

Ferroptosis is a newly recognized form of programmed cell death. The activation of the innate immune response can initiate ferroptosis in various types of cells. For instance, the activation of IFNR by binding with IFN‐γ leads to the activation of IRF1, which upregulates ACSL4 expression. When combined with arachidonic acid, IFN‐γ induces pronounced ferroptosis in various tumor cell lines.^[^ ^20a^ ^]^ The inhibition of toll‐like receptor 4 (TLR4) by a TLR4 inhibitor ameliorates ferroptosis in the hypoxic‐ischemic injury of hippocampal neurons.^[^ ^17a^ ^]^ IRF7 belongs to the transcription factors family of IRFs. Previous studies identified the role of IRF7 in transcriptionally activating IFN‐α/β expression, which resists infection by pathogens.^[^ ^27^ ^]^ In our study, we first found that TLR signaling and downstream IRF7 are involved in ferroptosis in BMSCs infected with *S. aureus* and *E. coli* by regulating ACSL4 expression, and IRF7 was the most upregulated transcriptional factor. However, the downstream pathways of TLR leading to IRF7 phosphorylation in *S. aureus*‐infected BMSCs (TRAF3‐IKKε/TBK1‐IRF7 axis) differ from those in *E. coli*‐infected BMSCs (MyD88‐IRAK4‐IRAK1/TRAF6‐IRF7 axis). This distinction might be due to the different bacterial components recognized by various TLRs in BMSCs. *E. coli* contains the classical TLR4 agonist, lipopolysaccharides (LPS), which can be recognized by TLR4 on the cell membrane, leading to MyD88‐dependent activation of Toll‐like receptor signaling.^[^ ^28^ ^]^ However, we also noticed that some downstream signal transducers of TLR3, including TRAF3, IKKε, and TBK1 could be activated by *E. coli*. This phenomenon was possibly caused by biological crosstalk in downstream of toll‐like signaling pathway, as the TLR4 downstream TRAM could activate TRAF3‐IKKε/TBK1 axis through TRIF.^[^ ^29^ ^]^ And the TRAF3‐IKKε/TBK1 axis may act as a signal bypass of *E. coli‐*activated IRF7. The classical upstream TLR for TRAF3 activation is TLR3, an intracellular receptor that recognizes double‐stranded RNA (dsRNA) from pathogens.^[^ ^30^ ^]^ Given that *S. aureus* secretes immunomodulatory RNA via membrane vesicles,^[^ ^31^ ^]^ we hypothesize that intracellular *S. aureus* may release dsRNA, triggering ferroptosis by activating TLR3 and downstream IRF7. However, this hypothesis remains to be further investigated and validated.

The importance of IRF7 in infection‐related immune response and tissue injury has recently garnered more attention. A recent study showed that self‐limited innate immune responses depend on the balance between IRF7 and IRF3. IRF3 inactivation leads to the overexpression of IRF7, exacerbating kidney pathology in *E. coli* infections. Conversely, IRF7 deletion protects mice from infection‐induced renal damage significantly.^[^ ^32^ ^]^ In our study, we observed a similar change in the IRF7/IRF3 ratio in RNA‐seq, with IRF7 upregulation and IRF3 downregulation. Our findings further elucidate the role of IRF7 in regulating ferroptosis by transcriptionally regulating ACSL4 expression, suggesting that IRF7 could be a promising therapeutic target to protect BMSCs from bacterial infections (as illustrated in the schematic diagram). Additionally, a notable upregulation HO‐1, encoded by *Hmox1*, was observed in both *S. aureus*‐infected and *E. coli*‐infected BMSCs. This phenomenon was consistent with the upregulated HO‐1 in macrophages infected by *M. tuberculosis*, which triggers ferroptosis in macrophages.^[^ ^33^ ^]^ Recent evidence supported that over‐activation of HO‐1 enhance intracellular Fenton reaction by increasing intracellular ferrous iron level thus facilitating ferroptosis.^[^ ^34^ ^]^ In this study, apparent upsurge of ferric iron level was found in *S. aureus*‐infected and *E. coli*‐infected BMSCs, which were supposed to be closely associated with upregulation of HO‐1. However, we found that upregulation of HO‐1 in BMSCs was not transcriptionally regulated by activating IRF7. Previous studies showed HO‐1 was mainly regulated by mitogen‐activated protein kinases (MAPK) signaling, PI3K‐Akt signaling, and Keap1/Nrf2 signaling.^[^ ^35^ ^]^ The exact mechanism underlying upregulation of HO‐1 in *S. aureus*‐infected and *E. coli*‐infected BMSCs remained elusive and need further investigation.

The limited availability of BMSCs and osteogenic dysfunction pose a challenge to infected bone defect repair. Past studies have intensively investigated the role of cytokines and inflammatory factors produced in the infection environment. The appropriate level of orchestrated inflammation was essential for neovascularization, BMSCs migration and osteogenic differentiation, thus facilitating bone regeneration in defect sites.^[^ ^1a^ ^]^ However, persistent activity of residual bacteria leads to altered cellular composition and cytokine profile, which exert obvious inhibitive effects on osteogenic differentiation.^[^ ^7d^ ^]^ Although these findings laid a strong foundation for understanding pathological progression in infected bone defects, the role of direct cytotoxicity of bacteria toward BMSCs in osteogenic dysfunction and bone repair was unclear due to unclarified death mechanisms of infected BMSCs. We first identified ferroptosis as the predominant death type of BMSCs infected with *S. aureus* and *E. coli*. Based on these findings, we used Fer‐1 to tentatively rescue osteogenic dysfunction caused by *S. aureus* and *E. coli* infections in vitro. The results demonstrated partially osteogenic‐rescuing effects of Fer‐1, with improved ALP expression, cell calcification and expression of important osteogenic transcription factors Runx2 and Osterix, which shed light on anti‐ferroptosis therapy for infected bone defects.

The repair of critical‐sized bone defects often necessitates the use of bone repair materials. Composite artificial bone scaffolds loaded with antibacterial components and bioactive factors have become a hotspot in the advances of orthopedic materials. For instance, a wood‐derived scaffold infused with chitosan quaternary ammonium salt (for antibacterial action) and dimethyloxalylglycine (pro‐osteogenic and pro‐angiogenic) has been designed for infected bone defect repair.^[^ ^36^ ^]^ Another innovation includes the 3D‐printed PLGA/Cu(I)@ZIF‐8 scaffold designed for infected bone repair.^[^ ^37^ ^]^ The 3D‐printed scaffold has shown huge potential in bone repair due to its adjustable internal structure and outstanding anatomical adaptability. However, most of these recently developed scaffolds tend to mechanically combine antimicrobial components with osteoinductive factors, often overlooking the injury‐mitigating effects on BMSCs in the infected sites. Additionally, these scaffolds frequently lack mechanisms for the responsive and sustained release of bioactive factors, hindering their adaptability to varying bacterial loads and sustained bone regeneration. Recently, phenylborate ester‐based ROS‐responsive biomaterials attracted researchers’ attention. The phenylborate ester bond is a type of ROS‐responsive covalent bond,^[^ ^38^ ^]^ and was used as a bridging segment connecting HACC and PEGDA‐DTT, the two polymer backbones in HACC‐DP/Fer‐1 hydrogel. When ROS levels increase in the infection environment in vivo or in the presence of H_2_O_2_ in vitro, the phenylborate ester bond was cleaved, leading to accelerated backbone breakage of hydrogel. Correspondingly, the release rate of encapsulated Fer‐1 in hydrogel was accelerated by ROS.

In this study, we constructed an antibacterial/anti‐ferroptosis 3D printed scaffold with ROS‐responsive releasing of HACC and Fer‐1 in the acute phase and long‐term releasing of Fer‐1 in the bone regeneration process. ROS‐responsive releasing of HACC and Fer‐1 from HACC‐DP hydrogel could adjust the antibacterial and ferroptosis‐rescuing effect according to the intensity of inflammation. And long‐term releasing of Fer‐1 from PCL/MBG/Fer‐1 scaffold could sustainably favor osteogenic differentiation. In vivo evaluations indicated that, while traditional treatments like bone wax and standard PCL/MBG scaffolds failed to effectively repair *S. aureus*‐infected bone defects, leading to pronounced osteolysis and pathological fractures, our newly developed scaffold demonstrated superior results. Specifically, the Fer‐1@HACC‐DP‐PCL/MBG scaffold, endowed with both antibacterial and anti‐ferroptosis properties, exhibited the most promising bone regeneration outcomes in infected defects. These findings suggest that targeting bacteria‐induced ferroptosis could significantly enhance bone regeneration in infected bone defects.

## 4 Conclusion

In conclusion, we identified ferroptosis as the predominant cell death of BMSCs infected with *S. aureus* and *E. coli* both in vitro and in vivo. Additionally, we ascertained that the osteogenic dysfunction of infected BMSCs can be effectively alleviated by inhibiting ferroptosis using Fer‐1. Stemming from these findings, we designed and developed a ROS‐responsive antibacterial/anti‐ferroptosis 3D‐printed bone repair scaffold. This scaffold is intended for the treatment of critical‐sized infected bone defects and has demonstrated robust antimicrobial properties and high efficiency in promoting bone regeneration in vivo. Moreover, we found that the TLR signaling pathway, along with its downstream component IRF7, plays a pivotal role in the initiation and execution of lipid peroxidation in BMSC ferroptosis by transcriptionally regulating ACSL4 expression.

## 5 Experimental Section

### Bacteria Culture

The *S. aureus* strain (ATCC 25923) and *E. coli* strain (ATCC 25922) were acquired from the American Type Culture Collection (ATCC). The *Xen 29* bioluminescent strain (IVISbrite 119240) was obtained from PerkinElmer. For the preparation of the bacterial inoculum, a fresh single bacterial colony was suspended in TSB and then incubated in a shaker at 37 °C and 200 rpm for 6 h.

### Murine Models of Acute Osteomyelitis and Implant‐Associated Infection

The murine acute osteomyelitis model and implant‐associated infection model were established according to previous studies.^[^ ^39^ ^]^ All operation procedures in the animal experiment had been reviewed and approved by the Animal Ethical Committee of the Shanghai Institute of Immunity and Infection, Chinese Academy of Sciences, with approval number No. A2023035. Adult male C57BL/6J mice (14‐week‐old) were purchased from Shanghai Jihui Laboratory Animal Company and were acclimated to the facilities for 3 days before the operation. For Fer‐1 pretreatment, mice were given a 10 mg Kg^−1^ administration (i.p. q.d.) for 3 days before the operation. To establish the acute osteomyelitis model, mice were first anesthetized with isoflurane. Then the skin near the knee joint was prepared and sterilized with a 75% ethanol solution. Next, 2 µL PBS or 10^6^ CFUs bacteria in 2 µl PBS were injected into the bone marrow cavity with a microsyringe. Compression hemostasis was performed, and all mice were carefully monitored until they awoke from anesthesia. On Day 3 post‐operation, the mice were sacrificed, and the bone marrow cells from the tibia were collected for further western blot (*n* = 4 per group) or flow cytometry (*n* = 6 per group) analysis. In addition, intratibial si‐Irf7 transfection was performed using an in vivo‐jetPEI siRNA delivery reagent (101000040, Polyplus, France) according to the instructions from the manufacturer. Ten micrograms si‐RNA (si‐Irf7) dissolved in the delivery reagent was intratibially injected 3 days before the bacteria injection. For the establishment of the murine implant‐associated infection model, procedures of animal preparation and anesthesia were the same as the above‐mentioned steps. Then an incision beside the femur was made with a scalpel. Soft tissues around the femur were carefully separated, and a small hole was made by a 29G needle at the middle of the femoral cortex. Next, a sterilized steel wire with a diameter of 0.3 and 4 mm length was immersed in the bacterial inoculum (10^8^ CFUs mL^−1^) and implanted in the bone marrow cavity of the femur by squashing the bacterial‐loaded wire into the hole on the femur. After this, the wound was sutured layer by layer, and animals were monitored until they awoke from anesthesia. On Day 7 post‐operation, mice were sacrificed, and the femur was isolated for further histological immunofluorescence staining (*n* = 3 per group).

### In Vivo Flowcytometry

After the mice were sacrificed, the tibias were isolated using sterile scissors and forceps. The bone marrow cells were rinsed with precooled FBS‐free RPMI 1640 culture medium and then filtered through a 70 µm cell filter (352350, BD Falcon, USA). After that, cell counting was performed and 2 × 10^6^ cells were harvested for further treatment and analysis. Next, cells were blocked with an anti‐CD16/32 antibody (1:300, 14‐0161‐86, eBioscience, USA) and then stained with an antibody cocktail containing PerCP/Cy5.5‐CD45 antibody (1: 300, 103131, BioLegend, USA), PerCP/Cy5.5‐CD31 antibody (1: 300, 102419, BioLegend, USA), PerCP/Cy5.5‐Ter119 antibody (1: 300, 116227, BioLegend, USA), and AF647‐LepR antibody (1 µg mL^−1^, 20966‐1‐AP, Proteintech, China, conjugated with AF647 using the FlexAble CoraLite Plus 647 Antibody Labeling Kit, Proteintech, China). The cells were then washed with FBS‐free RPMI 1640 medium and stained with the Liperfluo probe (1 µm, L248, DOJINDO, Japan). After staining, the cells were washed and subjected to flow cytometry analysis. At least 5 × 10^5^ cells were collected in each sample, and CD45^−^CD31^−^Ter119^−^LepR^+^ cells were gated as BMSCs. The median fluorescence intensity (MFI) of the FITC channel (Liperfluo) was analyzed. For intracellular IRF7 staining, the cells that were stained with CD45/CD31/Ter119/LepR were fixed with the Foxp3/Transcription Factor Staining Buffer Set (00‐5523‐00, eBioscience, USA) and subsequently stained with PE‐IRF7 antibody (1:300, 12‐5829‐82, eBioscience, USA). The cells were then washed and subjected to flow cytometry analysis.

### Cell Culture

Mouse BMSCs were isolated as previously described and expanded using the MesenCult™ Expansion Kit (05513, STEMCELL TECHNOLOGIES, Canada) according to the instructions from the manufacturer.^[^ ^40^ ^]^ In brief, bone marrow cells harvested from the femur and tibia of adult male C57BL/6J mice (14‐week‐old) were rinsed with MesenCult™ Expansion culture medium and then cultured in a culture plate at 37 °C in a 5% CO_2_ environment for 48 h. Then, the suspended cells were removed, and the culture medium was replaced every 2 days until passage. Purified P2 generation BMSCs were used for subsequent experiments.

### Bacteria‐BMSCs Co‐Culture Model

BMSCs (P2 generation) were seeded in 6‐well culture plates at a density of 5 × 10^5^ cells per well and cultured with α‐MEM culture medium (supplemented with 10% FBS without antibiotics) overnight at 37 °C, in a 5% CO_2_ incubator. For the pretreatment with death inhibitors, BMSCs were pretreated with the indicated concentrations of the inhibitors: Fer‐1 (10 µm), Lpx‐1 (1 µm), Nec‐1s (20 µm), and Z‐VAD‐FMK (50 µm) when the cells were seeded for 12 h. BMSCs were then treated with α‐MEM culture medium containing freshly activated *S. aureus* and *E. coli* with an MOI of 10 for the indicated time. After this, the bacteria were washed off, and the bacteria‐infected BMSCs were used for further analysis. The inhibitors Fer‐1 (S7243), Lpx‐1 (S7699), Nec‐1s (S8641), and Z‐VAD‐FMK (S7023) were purchased from Selleck (Selleck, China).

### C11‐BODIPY, Liperfluo, and FerroOrange Staining

C11‐BODIPY, Liperfluo, and FerroOrange staining were performed according to the instructions from the manufacturer. In brief, cells following the indicated stimulations were washed with Hanks' Balanced Salt Solution (HBSS, C0218, Beyotime, China) and subsequently incubated in working solutions of C11‐BODIPY (2.5 µm, D3861, Invitrogen, USA), Liperfluo (1 µm, L248, DOJINDO, Japan), and FerroOrange (1 µm, F374, DOJINDO, Japan). Finally, cells were washed and then observed using a confocal laser scanning microscope (CLSM, Leica, Germany). The intracellular lipid peroxides were determined by quantification of the fluorescence of C11‐BODIPY and Liperfluo, while the intracellular ferrous ions were determined by quantification of the fluorescence of FerroOrange.

### Live/Dead Staining Assay

The Live/Dead staining assay was performed using Live/Dead Fixable Dead Cell Stain Kits (L34970, Invitrogen, USA) and flow cytometry analysis. BMSCs following the indicated stimulation were stained with the reconstituted fluorescent reactive dye and fixed with 3.7% formaldehyde. The stained cells were then subjected to flow cytometry analysis. The dead cell rate was calculated and analyzed.

### Transmission Electron Microscopy

Transmission electron microscopy was performed as previously described.^[^ ^41^ ^]^ BMSCs after infection with *S. aureus* (MOI: 10) and *E. coli* (MOI: 10) for 12 h were fixed with 2.5% glutaraldehyde. The fixed cells were then subjected to gradient dehydration, followed by resin embedding and slicing. Then, samples were observed with transmission electron microscopy (TEM, FEI Talos L120C, Thermo Scientific, USA).

### Western Blot

Total cellular proteins were obtained by lysing cells with RIPA lysis (P0013B, Beyotime, China) containing a protease and phosphatase inhibitor cocktail (78445, Thermo Scientific, USA). Protein solutions were treated with an ultrasonic cell disruptor, and the protein concentration was quantified with a BCA assay. Each protein sample (20 µg) was separated by electrophoresis and transferred to a polyvinylidene fluoride membrane (0.22‐µm, Millipore, USA). The membrane was then blocked with 5% bovine serum albumin (BSA) solution and incubated with the primary antibody at 4 °C for 12 h. The next day, the membrane was washed with TBST and incubated in a secondary antibody at 4 °C for 2 h. The membrane was then imaged with a chemiluminescence imager (Touch Imager, e‐BLOT, China). The primary antibodies were as follows: 4‐HNE antibody (MAB3249, R&D SYSTEMS, USA), MDA antibody (NBP2‐59367, Novus, USA), ACTIN antibody (14395‐1‐AP, Proteintech, China), GPX4 antibody (ab125066, Abcam, UK), PTGS2 antibody (66351‐1‐Ig, Proteintech, China), HO‐1 antibody (10701‐1‐AP, Proteintech, China), ACSL4 antibody (ab155282, Abcam, UK), MyD88 antibody (23230‐1‐AP, Proteintech, China), IRAK1 antibody (D51G7, Cell Signaling Technology, USA), IRAK4 antibody (4363, Cell Signaling Technology, USA), TRAF6 antibody (ab 40675, Abcam, UK), TRAF3 antibody (33640, Cell Signaling Technology, USA), IKKε antibody (3416, Cell Signaling Technology, USA), TBK1 antibody (38066, Cell Signaling Technology, USA), Phospho‐TBK1 antibody (5483, Cell Signaling Technology, USA), IRF‐7 antibody (77 260, Cell Signaling Technology, USA), Phospho‐IRF‐7 (24129, Cell Signaling Technology, USA), ALPL Antibody (MA5‐24845, Invitrogen, USA), Collagen I antibody (66761‐1‐Ig, Proteintech, China), RUNX2 antibody (12556, Cell Signaling Technology, USA), Osterix antibody (ab209484, Abcam, UK).

### Cell Transfection

For IRF7 knockdown, BMSCs were transfected with siRNA targeting IRF7 using Lipofectamine RNAiMAX reagent (13778500, Thermo Scientific, USA) according to the instructions from the manufacturer. The siRNA sequences are listed in Table S1 (Supporting Information). For IRF7 overexpression, BMSCs were transfected with a mouse IRF7‐overexpression plasmid (54123) purchased from HanBio Co. Ltd (China) using Lipofectamine 3000 reagent (L3000075, Thermo Scientific, USA) according to the instructions from the manufacturer.

### RNA‐Sequencing

BMSCs were co‐cultured with *S. aureus* (MOI:10) and *E. coli* (MOI:10) for 12 h. The total RNA from both infected and uninfected BMSCs was extracted using a TRIzol reagent. The RNA samples were then sent to Sinotech Genomics Co. Ltd (Shanghai, China) for RNA‐sequencing analysis. A gene expression fold change (FC) ≥ 2‐fold and a Q value < 0.05 were considered indicative of differentially expressed genes.

### Immunofluorescence

BMSCs, after the indicated stimulation, were fixed with 4% paraformaldehyde and then permeabilized with 0.3% Triton X‐100 in PBS. The samples were then blocked with goat serum solution and incubated with primary ACSL4 antibody (ab155282, Abcam, UK) for 12 h. The next day, samples were washed with PBST and incubated with the secondary Anti‐rabbit IgG‐AF488 antibody (4412, Cell Signaling Technology, USA) for 2 h. Subsequently, cells were washed and stained with phalloidin for 40 min and observed using confocal laser scanning microscopy.

### ChIP Assay

ChIP assays were performed as previously described using a SimpleChIP Plus Enzymatic Chromatin IP Kit (9005, Cell Signaling Technology, USA).^[^ ^42^ ^]^ Chromatin from BMSCs was cross‐linked with 1% formaldehyde and then subjected to sonic treatment to produce ≈400 bp fragments. These fragments were immunoprecipitated with an anti‐IRF7 antibody (77260, Cell Signaling Technology, USA), normal IgG antibody (2729, Cell Signaling Technology, USA), and Histone H3 antibody (4620, Cell Signaling Technology, USA), and then pulled down by magnetic beads. The obtained DNA content was used for qRT‐PCR and agarose gel electrophoresis.

### Dual‐Luciferase Reporter Assay

The dual‐luciferase reporter assay was performed according to what was previously described.^[^ ^34b^ ^]^ In brief, the Acsl4‐WT‐Fluc reporter plasmid was constructed using a pGL4.10 vector. A pRLuc‐SV40‐N reporter plasmid (D2762) was purchased from Beyotime (China). For plasmid transfection in BMSCs, the Lipofectamine 3000 reagent was used (L3000075, Thermo Scientific, USA) according to the instructions from the manufacturer. For siRNA and plasmid co‐transfection, the Lipofectamine 2000 reagent (11668027, Thermo Scientific, USA) was used according to the instructions from the manufacturer. The BMSCs were subjected to bioluminescence detection 48 h post transfection.

### Osteogenic Induction Assay

BMSCs treated by the indicated stimulation were induced to osteogenic differentiation by osteogenic medium containing 10% FBS, 5 mm β‐Glycerophosphate disodium salt hydrate (G9422, Sigma‐Aldrich, USA), 50 µg mL^−1^ ascorbic‐acid (1043003, Sigma‐Aldrich, USA), 1 × 10^−7^ m dexamethasone (D4902, Sigma‐Aldrich, USA), 20 µg mL^−1^ Gentamicin (S4030, Selleck, China) and 10 µg mL^−1^ Oxacillin (S4974, Selleck, China). The osteogenic medium was replaced every 3 days. On Day 7 and Day 21 post‐induction, alkaline phosphatase staining and Alizarin Red S staining were performed, respectively.

### qRT‐PCR

qRT‐PCR was performed as previously described.^[^ ^43^ ^]^ In brief, total RNA from BMSCs was extracted with a TRIzol reagent. cDNA was reverse‐transcribed from the extracted RNA using a Script Reverse Transcription Kit (A2800, Promega, USA). qRT‐PCR was performed with SyBR Green qPCR Master Mix (B21202, Selleck, USA) on a Real‐Time PCR platform (QuantStudio 6, Thermo Scientific, USA). The 2^−ΔΔCt^ method was used to analyze the relative expression level of mRNA. Primer sequences are listed in Table S2 (Supporting Information).

### HACC‐PBA Synthesis

HACC with a substitution degree of 26% was synthesized according to the previous studies.^[^ ^44^ ^]^ In brief, 4 g chitosan (C105799, Aladdin, China) was dissolved in 200 mL 1% acetic acid solution and stirred for 3 h for complete dissolution. Then 7.5 g 3‐Glycidoxypropyldimethoxymethylsilane (G298766, Aladdin, China) was dissolved in 40 mL 1% acetic acid and dropwisely added to the chitosan solution. The solution was then stirred for reaction at 80 °C for 24 h. The reaction product was packed in a dialysis bag (molecular weight cut‐off of 3500) and subjected to dialysis in ddH_2_O for 72 h. After dialysis, the product was frozen at −80 °C and went through lyophilization to obtain the final HACC product. For HACC‐PBA synthesis, a specified amount of 4‐formylphenylboronic acid was added to a solution of HACC (200 mg mL^−1^) dissolved in deionized water. The mixture was stirred for 1 h at room temperature. Subsequently, an amount of NaBH3CN equivalent to three moles was added to the solution and the mixture was stirred for an additional 20 h. Finally, the product, PBA‐modified HACC, underwent dialysis and freeze‐drying. The resulting product was collected for further hydrogel synthesis.

### Preparation of ROS‐Responsive Antimicrobial/Anti‐Ferroptosis Hydrogel

The antimicrobial/anti‐ferroptosis hydrogel was formulated utilizing a dual‐cross‐linking system that combines the photo‐cross‐linking of PEGDA‐DTT polymers with the covalent cross‐linking between HACC‐PBA and PEGDA‐DTT. Initially, PEGDA‐DTT was prepared by mixing DTT (100 mg mL^−1^) with PEGDA (150 mg mL^−1^) at a specified ratio for 2 h, as detailed in the prior study.^[^ ^45^ ^]^ Subsequently, HACC‐PBA (ranging from 0% to 1.5%), Fer‐1 (to achieve a final concentration of 20 mg mL^−1^), and 0.5% LAP were introduced into the PEGDA‐DTT solution. This mixture was stirred for 20 min. Once homogenously mixed, the solution was poured into molds or applied onto 3D‐printed PCL/MBG/Fer‐1 scaffolds. The final step involved photo‐cross‐linking the HACC‐DP/Fer‐1 hydrogel by exposing it to blue light (405 nm) for 20 s.

### Characterization

The synthesis of HACC and HACC‐PBA was characterized using both FTIR and NMR spectroscopy. FTIR spectra were obtained using an FT‐IR Spectrometer (FT/IR‐4600, JASCO, Japan). Meanwhile, NMR spectra were recorded using an NMR spectrometer (Bruker‐400 MHz, Bruker, USA). To assess the rheological properties of the HACC‐DP hydrogel, measurements were taken using a HAAKE rheometer (Thermo Scientific, USA) with 1% strain and 37 °C. To assess the shear‐thinning property of HACC‐DP hydrogel, the uncross‐linked hydrogel was subjected to a rheological test with a shear rate from 0.1–100 (1/s) at 37 °C. The compression modulus of the scaffold was measured using an electronic universal testing machine (5982, INSTRON, USA).

### Scanning Electron Microscopy

The morphology of bacteria treated with different scaffolds was observed with scanning electron microscopy. In brief, *S. aureus* inoculum was freshly activated by overnight shaking and diluted to 10^7^ CFUs mL^−1^ in TSB. Then, different scaffolds were incubated in 100 µL bacteria inoculum at 37 °C for 6 h. The scaffolds were then fixed with 2.5% glutaraldehyde and subjected to gradient dehydration. The dehydrated scaffolds were coated with gold for observation under a scanning electron microscope (S4800, HITACHI, Japan).

### Construction of Antimicrobial/Anti‐Ferroptosis 3D‐Printed Scaffold for Bone Repair

PCL/MBG/Fer‐1 scaffolds were fabricated following the previously established protocols.^[^ ^46^ ^]^ Initially, a mixture powder of MBG and Fer‐1 was prepared. MBG and Fer‐1 were combined in a 7:1 mass ratio and then lyophilized to form the composite powder. PCL was dissolved in methylene chloride, and either the MBG/Fer‐1 composite or just MBG was introduced at a 4:9 mass ratio (either PCL: MBG/Fer‐1 or PCL:MBG). This blend was stirred thoroughly for 2 h. After allowing the organic solvent to partially evaporate, the uniform mixture was loaded into a 3D printer (Biobuild‐S, Regenovo, China). 3D‐printing was conducted using the following parameters: temperature = 25 °C, the diameter of nozzle = 0.25 mm, speed = 1.8 mm s^−1^, pressure = 3.0 bar, the distance between strips = 500 µm, the diameter of layer = 3 mm, scaffold height = 4 mm. After printing, the scaffold was allowed to fully dry to ensure complete evaporation of the organic solvent. Once dried, the scaffold was incorporated with the HACC‐DP/Fer‐1 hydrogel. This hydrogel was then photo‐cross‐linked under the illumination of blue light (405 nm) for 20 s.

### Releasing and Degradation of HACC‐DP Hydrogel and PCL/MBG 3D‐Printed Scaffold

Release and degradation patterns of Fer‐1 in HACC‐DP hydrogel and PCL/MBG scaffold were simulated by measuring the release of FITC in these two systems. In brief, HACC‐DP/FITC hydrogel and PCL/MBG/FITC scaffold were prepared with the same content of FITC as Fer‐1. HACC‐DP/FITC hydrogel was immersed in SBF or SBF containing 100 µm H_2_O_2_ (volume ratio of HACC‐DP/FITC:SBF = 1:500). PCL/MBG/FITC scaffold was immersed in SBF or SBF containing 100 µm H_2_O_2_ with a ratio of 1 g:100 mL. At predetermined time points, the supernatants were collected and stored at −20 °C in the dark until further analysis. Hydrogel and scaffold were washed with ddH_2_O and dried by lyophilization, and the mass was determined by an electronic balance. The release amount of FITC was calculated using a standard fluorescence‐concentration curve of FITC (Figure S4F, Supporting Information).

### Antimicrobial Assay

The antimicrobial assay was performed as previously described.^[^ ^47^ ^]^ In brief, *S. aureus* (ATCC 25923) was freshly activated by suspending a single bacteria colony in TSB broth. After incubation at 37 °C for 3, 6, and 24 h with shaking, the bacterial concentration was counted and diluted to 10^6^ CFUs mL^−1^. Then, antimicrobial/anti‐ferroptosis 3D‐printed scaffolds containing different concentrations of HACC‐PBA in loaded HACC‐DP hydrogels were immersed in 100 µL of 1 × 10^6^ CFU mL^−1^ bacterial suspension and incubated at 37 °C for indicated periods. After that, 1 mL of sterile PBS was added into the tube and it was subjected to ultrasonic oscillation to sufficiently resuspend the bacteria. Serial dilution and drop plate methods were performed and the number of colonies was counted. All groups were normalized to the Ctrl group (TSB) in the loaded hydrogel.

### CCK‐8 Assay

The cell viability of BMSCs seeded on Fer‐1@HACC‐DP‐PCL/MBG scaffolds was measured by CCK‐8 assay (Beyotime Biotechnology, China) according to the manufacturer's instructions. In brief, 2 × 10^4^ BMSCs were seeded on Fer‐1@HACC‐DP‐PCL/MBG scaffolds in 48‐well culture plates. The cell viability of BMSCs was measured by OD_450_ on Day 1, 4, and 7.

### Rat Model of Distal Femur Critical‐Sized Infected Bone Defect

All operation procedures in the animal experiment had been reviewed and approved by the Animal Ethical Committee of the Shanghai Institute of Immunity and Infection, Chinese Academy of Sciences, with approval number No. A2023035. Adult male SD rats (250–300 g per rat) were obtained from the Charles River Animal Company. The rats were accustomed to breeding facilities for 4 days. On the day of the operation, rats were anesthetized with isoflurane. The skin near the knee joint was prepared and sterilized using a 75% ethanol solution. Next, a longitudinal incision was made on the inner side of the joint midline. The joint capsule was opened and the medial condyle of the femur was exposed. Then, a round defect with a diameter of 3 mm and a depth of 4 mm was made on the lateral side of the medial condyle using a trephine. After that, 50 µL of 2 × 10^8^ CFUs mL^−1^ *S. aureus* (*Xen 29*) suspension was injected into the defect sites. Bone wax or scaffolds were then implanted into defect sites. The wound was sutured layer by layer, and animals were monitored until they awakened from anesthesia. The animals were randomly designated to four groups: Bone wax group (control group), PCL/MBG scaffold group (without antibacterial/anti‐ferroptosis properties), HACC‐DP‐PCL/MBG scaffold (antibacterial scaffold), and Fer‐1@HACC‐DP‐PCL/MBG scaffold (antibacterial/anti‐ferroptosis scaffold). Each group contained 12 rats, with 6 rats sacrificed at Week 6 and 6 rats sacrificed at Week 12 post‐operation. At predetermined time points, IVIS was performed to monitor bacterial burden. Rats were separately sacrificed at Week 6 and Week 12. After that, the isolated femur was fixed with 4% paraformaldehyde solution and stored at 4 °C for further analysis.

### Rat Model of Subcutaneous Infection

All operation procedures in the animal experiment had been reviewed and approved by the Animal Ethical Committee of the Shanghai Institute of Immunity and Infection, Chinese Academy of Sciences, with approval number No. A2023035. Adult male SD rats (200 – 300 g per rat) were obtained from the Charles River Animal Company. The rats were accustomed to breeding facilities for 4 days. The HACC‐DP‐2/Fer‐1 hydrogel and HACC‐DP‐0/Fer‐1 hydrogel were photo‐cross‐linked in a 48‐well plate with a volume of 150 µl. On the day of the operation, rats were anesthetized with isoflurane. The skin on the back of the rat was prepared and sterilized with 75% ethanol. Then a longitudinal incision was made with sterile ophthalmic scissors, and a subcutaneous cavity was made by blunt dissection. In the uninfected HACC‐DP‐2/Fer‐1 hydrogel group, HACC‐DP‐2/Fer‐1 hydrogel was implanted in the subcutaneous cavity and 50 µL PBS was injected into the cavity. In infected groups, HACC‐DP‐2/Fer‐1 hydrogel or HACC‐DP‐0/Fer‐1 hydrogel was implanted in the subcutaneous cavity, and 50 µL 2 × 10^8^ CFUs mL^−1^ *S. aureus* (*Xen 29*) was injected into the cavity. The wound was sutured layer by layer, and animals were monitored until they awakened from anesthesia. Each group contained 3 rats. Rats were sacrificed on Day 14 post‐operation and then the skin tissue on the back was separated and fixed in 4% paraformaldehyde. The tissue was then subjected to histological evaluation.

### X‐Ray and Micro‐CT Analysis

X‐ray radiography of femur from rats was performed using a small animal X‐ray imager (MX20, Faxitron Bioptics, USA). Micro‐CT scanning and 3D image reconstruction were performed using a Micro‐CT system (µCT80, Scanco Medical, Switzerland).

### Histological Analysis

Histological analysis was performed as previously described.^[^ ^48^ ^]^ In brief, rat specimens fixed with 4% paraformaldehyde were decalcified for 2 months and then underwent paraffin embedding and sectioning. Tissue sections, 5 µm thick, were then stained with Masson trichrome, Giemsa, and immunofluorescence (IF) staining. The primary antibodies used for IF were listed as follows: OCN (23418‐1‐AP, Proteintech, China), LepR (20966‐1‐AP, Proteintech, China).

### Statistical Analysis

Independent experiments or repeated measurements were conducted for all data (*n* = 3, 4, or 6, respectively). All data were presented as mean ± standard deviation (SD). A *P* value less than 0.05 was considered statistically significant. Comparisons between the two groups were conducted using a two‐tailed Student's *t*‐test. Multiple comparisons were conducted using one‐way ANOVA followed by Tukey's post hoc test. For multivariate parametric data, two‐way ANOVA followed by Tukey's post hoc test was used. Analysis was conducted using Graphpad Prism Software (Version 8.0, Boston, USA).

## Conflict of Interest

The authors declare no conflict of interest.

## Author Contributions

K.Y., Y.Y., and Y.L. contributed equally to this work. K.Y., Y.Y., and Y.L. designed and performed the experiments and analyzed the data. K.Y., S.Y., H.L., Y.L., and T.T. conceived and designed the overall study as principal investigators. F.Z., Y.L., K.H., W.K., F.L., T.K., Y.W., C.C., H.A., and Z.Y. performed the experiments and analyzed the data. H.C. and Y.L. contributed to the transmission electron microscopy imaging and analysis. J.H. contributed to the analysis of flow cytometry. All authors contributed to the writing and editing of the manuscript and approved the final manuscript.

## Supporting information

Supporting Information

## Data Availability

The data that support the findings of this study are available from the corresponding author upon reasonable request.
