# Supplementary material for: Targeting Bacteria‐Induced Ferroptosis of Bone Marrow Mesenchymal Stem Cells to Promote the Repair of Infected Bone Defects
Source: Adv Sci (Weinh). 2024 Aug 21;11(39):2404453. doi: 10.1002/advs.202404453 (PMC11497072; doi:10.1002/advs.202404453)
Supplement: Supplementary file 1 — Supporting Information [file ADVS-11-2404453-s001.docx]

**Supporting information**

**Targeting Bacteria-Induced Ferroptosis of Bone Marrow Mesenchymal Stem Cells to Promote the Repair of Infected Bone Defects**

*Kai Yuan, Yiqi Yang, Yixuan Lin, Feng Zhou, Kai Huang, Shengbing Yang, Weiqing Kong, Fupeng Li, Tianyou Kan, Yao Wang, Caiqi Cheng, Yakun Liang, Haishuang Chang, Jie Huang, Haiyong Ao, Zhifeng Yu, Hanjun Li*, Yihao Liu*, Tingting Tang**

**Figure S1. Representative Live/Dead staining images of *S. aureus-infected* and *E. coli-infected* BMSCs.**

**Figure S2. Antibacterial effects of different cell death inhibitors.**

**Figure S3. Principal component analysis (PCA) analysis and qRT-PCR analysis.**

**Figure S4. Characterization of HACC-DP hydrogel and Fer-1@HACC-DP-PCL/MBG scaffold.**

**Figure S5. In vivo degradation and antibacterial effects of HACC-DP-2 hydrogels in rat subcutaneous infection model.**

**Figure S6. Representative X-ray images of the femur from rats in each group at Week 6 and Week 12.**

**Table S1. Sequences of siRNA used in cell transfection.**

**Table S2. Sequences of primers used in qRT-PCR.**

**Table S3. Sequences of primers used in ChIP-PCR.**

**Supplementary figures**


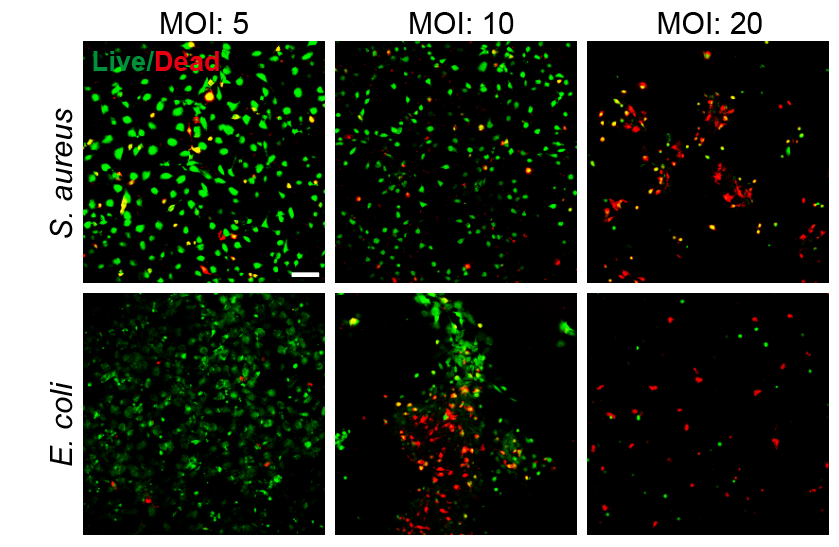


**Figure S1. Representative Live/Dead staining images of *S. aureus-infected* and *E. coli-infected* BMSCs.** BMSCs were infected with *S. aureus* and *E. coli* with MOI ranging from 5 to 20 for 12 h. Scale bar = 80 μm.


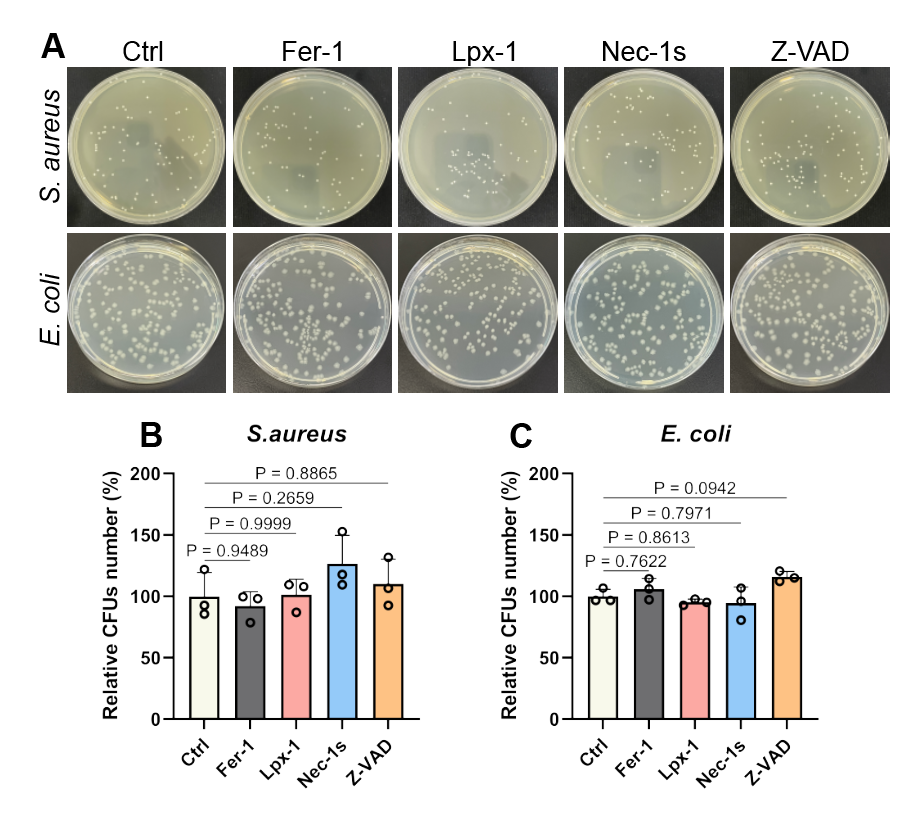


**Figure S2. Antibacterial effects of different cell death inhibitors.** (A) Representative images of CFUs counting. S. aureus and E. coli inoculum with 5.5×10^5^ CFUs/ml were treated by Fer-1 (10 μM), Lpx-1 (1μM), Nec-1s (20 μM), and Z-VAD-FMK (50 μM) for 20 h. Then serial dilution and spreading plate were performed to calculate CFUs number. (B-C) Quantitative analysis of CFUs number relative to Ctrl group in (A). Values are means ± SDs (*n* = 3). Multiple comparison was performed by one-way analysis of variance (ANOVA) with Tukey’s post-hoc analysis.


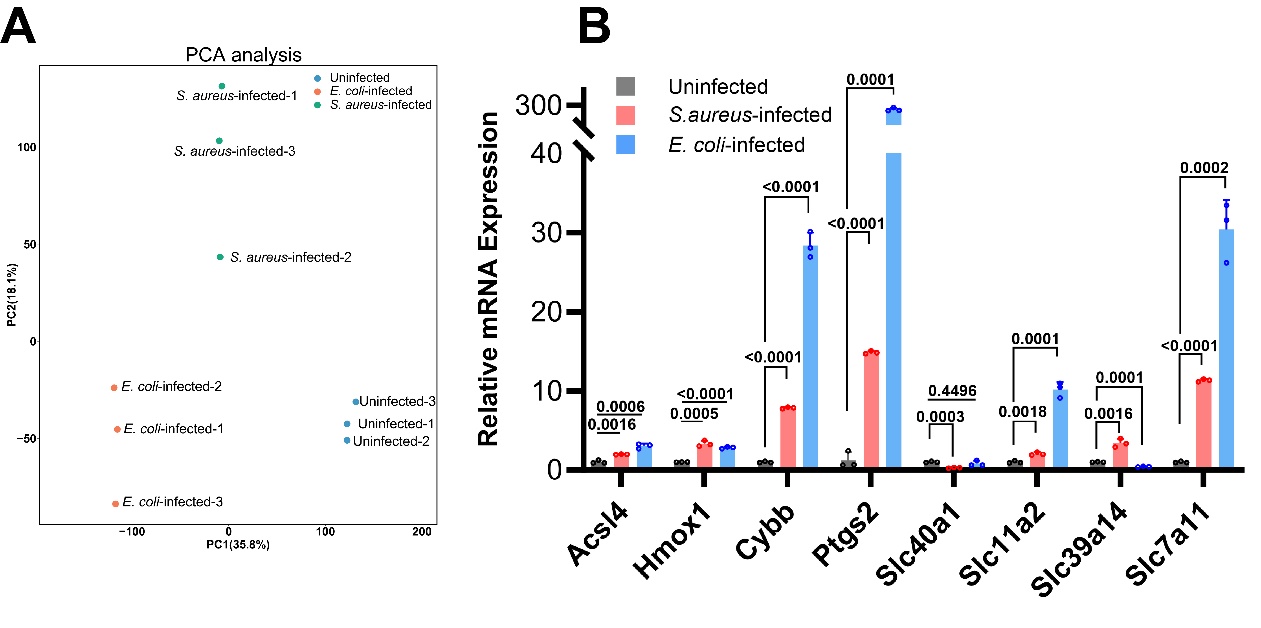


**Figure S3. Principal component analysis (PCA) analysis and qRT-PCR analysis.** (A) PCA analysis of differentially expressed genes in three groups in RNA-seq. (B) Gene expression of ferroptosis-related genes was measured by qRT-PCR. The *Hsp90b1* gene was used as an internal reference gene. Values are means ± SDs (*n* = 3). Comparison between two groups (*S. aureus*-infected vs Uninfected and *E. coli*-infected vs Uninfected) was performed by two-tailed Student’s *t-test*.


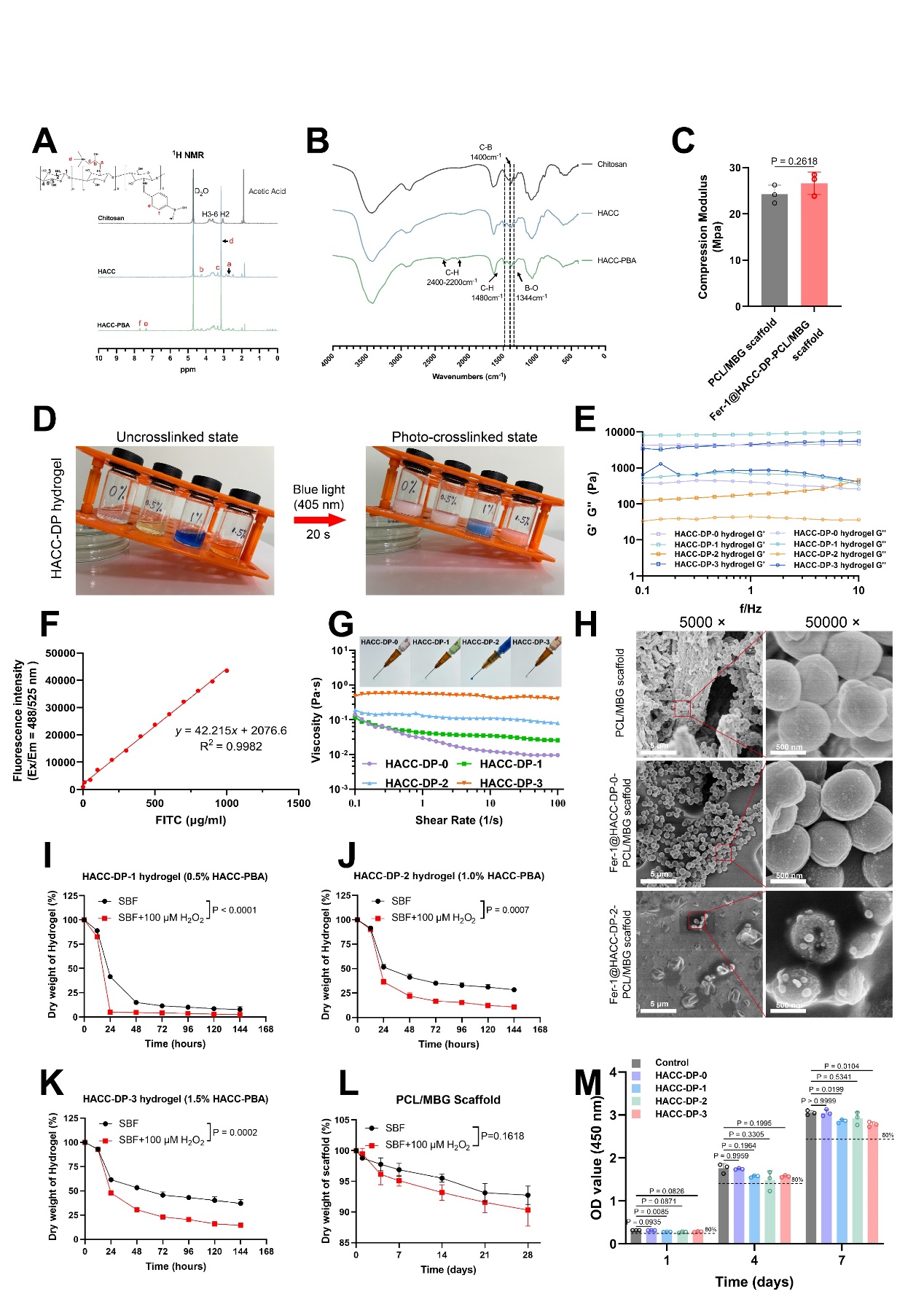


**Figure S4. Characterization of HACC-DP hydrogel and Fer-1@HACC-DP-PCL/MBG scaffold.** (A) ^1^H NMR spectrum of Chitosan, HACC, and HACC-PBA. (B) FTIR spectra of Chitosan, HACC, and HACC-PBA. (C) Compression modulus of PCL/MBG scaffold and Fer-1@HACC-DP-PCL/MBG scaffold. Values are means ± SDs (*n* = 3). Statistical analysis between groups was performed by a two-tailed Student’s *t-test*. (D) Images of un-crosslinked and crosslinked HACC-DP hydrogel containing 0% (HACC-DP-0), 0.5% (HACC-DP-1), 1.0% (HACC-DP-2), 1.5% HACC-PBA (HACC-DP-3) content. Un-crosslinked HACC-DP hydrogel was subjected to blue light (405 nm) irradiation for 20s and then got photo-crosslinked. (E) Frequency-depended rheological performance of HACC-DP hydrogel at the strain of 1%. (F) Standard curve of FITC concentration versus FITC fluorescence upon excitation and emission of Ex/Em = 488/525 nm. A linear regression equation was calculated. (G) The shear-thinning effect and injectability of HACC-DP hydrogels. (H) Representative images of scanning electron microscopy of *S. aureus* on different scaffolds. Scale bar = 5 μm in low magnification images (5000×). Scale bar = 500 nm in high magnification images (50000×). Note: The rectangular dark marks in the image of Fer-1@HACC-DP-2-PCL/MBG scaffold (5000×) were caused by rectangular electron beam-induced damage to the hydrogel polymer, which occurred during high magnification imaging (50000×). (I-K) Weight loss of HACC-DP hydrogel containing 0.5%, 1.0% and 1.5% HACC-PBA content after degradation in SBF and SBF with H_2_O_2_ (100 μM) at different time points. A significantly higher degradation rate observed in groups of HACC-DP-2 hydrogel and HACC-DP-3 hydrogel in SBF with H_2_O_2_ (100 μM) compared with that in SBF indicated obvious ROS-responsive release capability of HACC-DP hydrogel. Values are means ± SDs (*n* = 3). Two-way analysis of variance (ANOVA) was performed. (L) Weight loss of PCL/MBG/FITC scaffold after degradation in SBF and SBF with H_2_O_2_ (100 μM) at different time points. No significant difference was observed in degradation rates between scaffolds in SBF and SBF with H_2_O_2_ (100 μM). Values are means ± SDs (*n* = 3). Two-way analysis of variance (ANOVA) was performed. (M) Cell viability of BMSCs seeded on culture disk (control), Fer-1@HACC-DP-/PCLMBG scaffolds (loaded with HACC-DP-0 hydrogel, HACC-DP-1 hydrogel, HACC-DP-2 hydrogel, and HACC-DP-3 hydrogel) from 1day to 7days. Cell viability of BMSCs in different HACC-DP hydrogel groups was above 80% compared with the control group. Values are means ± SDs (*n* = 3). Multiple comparison was performed by one-way analysis of variance (ANOVA) with Tukey’s post-hoc analysis.


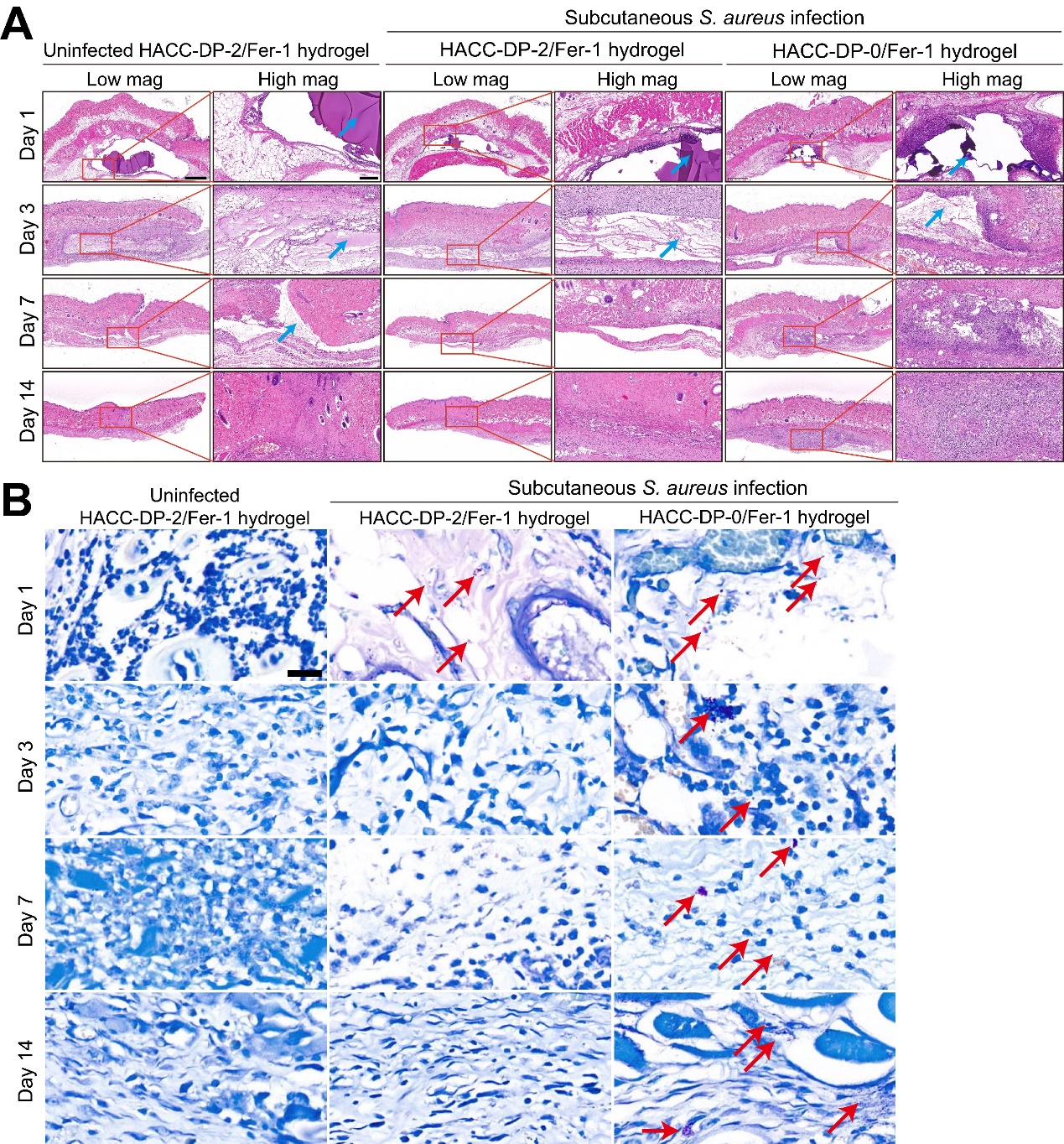


**Figure S5. In vivo degradation and antibacterial effects of HACC-DP-2 hydrogels in rat subcutaneous infection model.** (A) Representative images of H&E staining from skin tissue of rats in each group from Day 1 to Day 14 post operation. Scale bar = 1 mm (low magnification). Scale bar = 200 μm (high magnification). Hydrogels were marked with blue arrow. (B) Representative images of Giemsa staining from skin tissue of rats in each group from Day 1 to Day 14 post operation. Scale bar = 25 μm. Bacterial colonies were labelled with red arrow. Each group included 3 rats (*n* = 3).


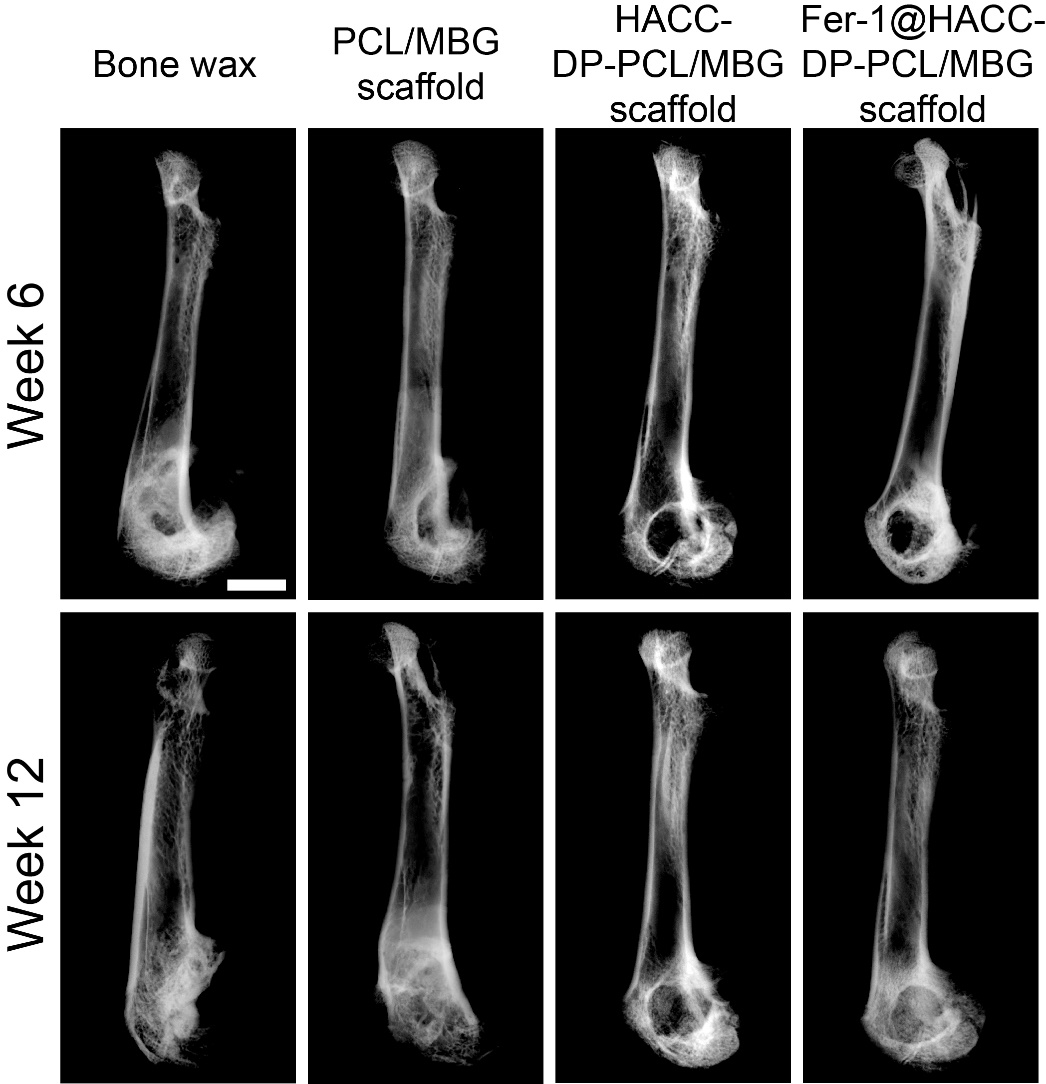


**Figure S6. Representative X-ray images of the femur from rats in each group at Week 6 and Week 12.** Scale bar = 3 mm.

**Table S1. Sequences of siRNA used in cell transfection**

| si-RNA | Sequence (5’-3’) |  |
| --- | --- | --- |
| si-Irf7-1 | Forward | GGAUCUGGAUGAAGAAGAUTT |
|  | Reverse | AUCUUCUUCAUCCAGAUCCTT |
| si-Irf7-2 | Forward | GCACUUUCUUCCGAGAACUTT |
|  | Reverse | AGUUCUCGGAAGAAAGUGCTT |
| si-Irf7-3 | Forward | CCCUCUGCUUUCUAGUGAUTT |
|  | Reverse | AUCACUAGAAAGCAGAGGGTT |
| Negative Ctrl | Forward  Reverse | UUCUCCGAACGUGUCACGUTT  ACGUGACACGUUCGGAGAATT |

**Table S2. Sequences of primers used in qRT-PCR**

| Gene | Primer Sequence (5’-3’) | |
| --- | --- | --- |
| *Cybb* | Forward | TGATAAGCAGGAGTTCCAAGAT |
|  | Reverse | ACTAACATCACCACCTCATAGC |
| *Ptgs2* | Forward | CCAGCAGACTCATACTCATAGG |
|  | Reverse | GAAGTGGTAACCGCTCAGG |
| *Slc11a2* | Forward | TTCTTATGAGCATTGCCTACCT |
|  | Reverse | GGTGACCACTCCAAGTCTC |
| *Slc7a11* | Forward | TGCAAGCTCACAGCAATTCT |
|  | Reverse | CCATTAGACTTGTGTCTCTTCC |
| *Irf7* | Forward | AAGGCATCACAGAGTAGTAGCA |
|  | Reverse | CCAATAGCCAGTCTCCAAACAG |
| *Acsl4* | Forward | GATGATTGCAGCACAGACTTG |
|  | Reverse | CAGATAGGAAGCCTCAGATTCA |
| *Hmox1* | Forward | AAGCCGAGAATGCTGAGTTCA |
|  | Reverse | GCCGTGTAGATATGGTACAAGGA |
| *Hsp90b1* | Forward | GCTGAAGTGAACAGGATGATGA |
|  | Reverse | GGAGATGAGCCTTATCTTGTCT |

**Table S3. Sequences of primers used in ChIP-PCR**

| si-RNA | Sequence (5’-3’) |  |
| --- | --- | --- |
| Binding site 1 | Forward | CCGAGTGAGCAGGGAATCTT |
|  | Reverse | TGCCTCGTGCTTCCTATGG |
| Binding site 2 | Forward | CGGAGTGCTGGGATTAAAGG |
|  | Reverse | TGCTTTAGGCTTTGTGTGTCA |
| Binding site 3 | Forward | GCTTGGGCTAACAGGTTGAT |
|  | Reverse | CCATCTATTCCTCCAACAAACC |
